# Menstrual cycle phase and its association with COVID-19 vaccine outcomes among period tracking app users

**DOI:** 10.1038/s44294-026-00150-x

**Published:** 2026-06-06

**Authors:** Poppy Alexandra Cooper, Emily R. Boniface, Blair G. Darney, Alison Edelman, Rebecca Sear, Amanda A. Shea, Sarah Walters, Kirsten Weber, Virginia J. Vitzthum, Alexandra Alvergne

**Affiliations:** 1 LSHTM (London School of Hygiene & Tropical Medicine), London, UK; 2 OHSU (Oregon Health & Science University), Portland, OR USA; 3 Brunel University, London, UK; 4 Clue (BioWink GmbH), Berlin, Germany; 5 Centre for Menstrual Cycle and Ovulation Research (CeMCOR), Department of Medicine, University of British Columbia, Vancouver, BC Canada; 6 Indiana University, Bloomington, IN USA; 7 ISEM (Institute of Evolutionary Science of Montpelier), University of Montpellier, CNRS, IRD, Montpelier, France

**Keywords:** Diseases, Health care, Immunology, Medical research, Risk factors

## Abstract

Despite known hormonal influences on immune function, the impact of menstrual cycle phase on vaccine outcomes remains unexplored. Using prospectively tracked cycle data from the Clue period tracking-app, matched with an in-app survey of 1474 women, we compared self-reported COVID-19 vaccine outcomes between follicular-phase (estrogen-dominant) and luteal-phase (progesterone-dominant) vaccinated women. Side effect presence, severity and number were analysed using binary, ordinal, and negative binomial regression respectively, and post-vaccination time to infection (67–372 days) via Wilcoxon rank-sum test. Follicular-phase vaccination was associated with 35% higher odds of any self-reported side effects (OR: 1.35, 98.3% CI: 1.01–1.79) compared to luteal-phase vaccination. Median time to infection was 35 days longer following follicular-phase vaccination (200 [140–237] vs 165 [107–210] days, *p* = 0.05), though infection numbers were limited. These findings suggest menstrual cycle phase warrants consideration in sex-based immunity research and may inform future research on vaccination and personalised health strategies.

## Introduction

The coincidence of the COVID-19 pandemic with the rise of big data on menstrual cycles has enabled a substantial leap in studying the previously overlooked relationship between menstrual cycles and vaccine response^1,2^. Recent research has explored whether COVID-19 and flu vaccinations associate with changes to cycle length, bleeding intensity, and pain^3–10^. Several studies have found significant, albeit small and transient, changes with no lasting fertility effects^11^, while others have found no objective alterations to menstrual cycle parameters following vaccination^12^. Prospectively collected data from period-tracker apps indicate that the observed changes in menstrual cycle parameters following vaccination may be driven by vaccination during the follicular phase^8,13,14^, reflecting the tight connection between endocrine and immune function^1,15^. To date, the reverse relationship—whether the menstrual cycle and its fluctuating hormones influence vaccine outcomes—remains unexplored. This paper builds on this growing interest in menstrual-vaccine interactions, by examining whether timing of vaccination within the menstrual cycle is associated with subsequent vaccine-induced side effects or break-through infection risk.

Sex differences in vaccine efficacy and side effects have been extensively documented, with these disparities evident even before puberty^16–22^. Women typically mount stronger humoral and cellular responses to infection and vaccination, and also report more adverse side effects than men^17^. Indications of sex differences in COVID-19 immunity include greater morbidity and mortality in men^23–25^ but more frequent and severe vaccine side effects in women^26,27^, who also have a higher prevalence of long COVID-19^28^. These variations in immune response are linked to behavioural, genetic, and reproductive hormone factors^29–31^, with reproductive hormones particularly explaining the increased disparity observed during reproductive years^18,21,32^.

Sex steroid hormones, estradiol (E2) and progesterone (P4), fluctuate over the menstrual cycle, creating additional immune variation beyond baseline sex differences^15^. While context dependent, estradiol generally enhances immune reactivity in a dose dependent manner^33^, while progesterone tends to suppress it^34,35^, creating dynamic variations in immune system functionality across cycle phases. These hormones modulate immune responses through receptors on immune cells including neutrophils, dendritic cells, macrophages and lymphocytes^18,32,36^. These effects contribute to cycle-dependent variations in susceptibility to infections^37,38^, manifestations of autoimmune diseases^39–42^, variation in immune markers of inflammation such as CRP^43,44^ and total leucocyte count^45^, as well as specific changes in natural killer, T and B cell responses^46–48^. Given these hormonal influences on immune function, we can hypothesise how different menstrual cycle phases might affect vaccine responses. The follicular phase—with rising estrogen levels and the inflammatory event of menstruation—may enhance both vaccine side effects (reactogenicity) and protection from infection (immunogenicity), while the progesterone-dominant luteal phase may dampen them.

Clarifying the relationship between menstrual cycle phase at vaccination and subsequent vaccine response therefore represents an exciting opportunity to advance personalised medicine, enhancing vaccine effectiveness and individual health outcomes. The transient menstrual disturbances reported following COVID-19 vaccination have raised concerns that could fuel vaccine hesitancy^2^ making this research particularly timely amid growing recognition of the urgent need for sex-specific and cycle-aware medical research^15,16,49–53^.

Cycle-tracking apps have proven to be powerful tools in menstrual health research, enabling researchers to gather large real-world datasets^9,10,54–58^. Specifically, this study leverages data from the cycle-tracking app Clue to examine whether menstrual cycle phase at the time of first COVID-19 vaccination is associated with vaccine side effects and risk of subsequent infection. Using an integrated dataset of 1474 individuals who completed a vaccine-focused survey and prospectively-logged menstrual data, we tested two primary hypotheses in relation to COVID-19 vaccination: (1) vaccine reactogenicity, measured by the presence, number, and severity of side effects, will be greater when vaccination occurs during the follicular phase; and (2) post-vaccination COVID-19 infection risk will be lower when vaccination occurs during the follicular phase. These hypotheses are based on the fact that the follicular phase is characterised by rising estrogen—an immune enhancer—whereas the luteal phase is dominated by progesterone, which can act as an immune suppressant^32^.

## Results

### Study population

**Fig. 1:**
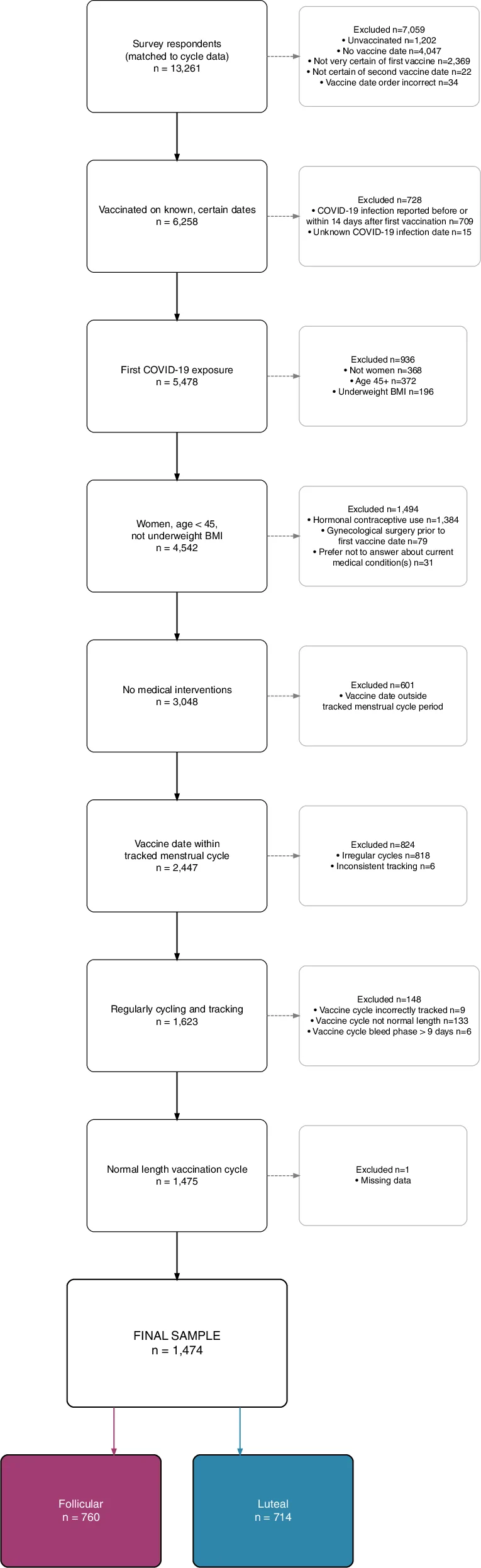
Study sample selection. Flowchart showing data exclusion steps and final sample size. *n* = 13,261 survey respondents were matched to tracked cycle data. Unvaccinated individuals, those with missing vaccine dates or with infection dates prior to their first COVID-19 vaccine were excluded. Anyone age 45 + , who did not identify as a woman or who had an underweight BMI was excluded, as well as anyone using hormonal contraceptives at the time of vaccination and anyone who reported gynaecological surgery prior to their COVID-19 vaccination. Participants whose vaccination date fell outside of a tracked menstrual cycle period were excluded, as were those with irregular cycles, inconsistent tracking, or whose cycle or menstruation length fell outside of the FIGO (International Federation of Gynaecology and Obstetrics) definition of regular (cycle length = 24–38 days, or menstruation < 9 days⁶⁰). One participant was excluded for missing data. A final sample of *n* = 1474 was included for analysis, of which *n* = 760 reported first COVID-19 vaccination in the follicular phase and *n* = 714 in the luteal phase. BMI = body mass index.

Following exclusions, 1474 participants were included for analysis: 760 vaccinated during the follicular phase and 714 during the luteal phase (Fig. 1). Using prospectively tracked cycle data, we defined the 14 days preceding the onset of menstruation as the luteal phase. Any remaining days in the cycle were assigned to the follicular phase. The analysis was restricted to cycles 24–38 days long (as per FIGO criteria for a regular cycle^59^, (see *Methods* for further details) with variation in cycle length being due to the follicular phase. Participant characteristics by vaccination cycle phase are detailed in Table 1. The two groups showed no significant differences in demographic characteristics, health behaviours, vaccination details, or survey timing. Cycle length differed slightly, with those vaccinated in the follicular phase having a 1 day longer median cycle length (29, IQR (27–31)) than those vaccinated in the luteal phase (28, IQR (27-30)).

**Table 1:** Participant characteristics by menstrual cycle phase (follicular/luteal) at first COVID-19 vaccination

Characteristic	Overall *N* = 1474^a^	Luteal *N* = 714^a^	Follicular *N* = 760^a^	*p*-value^b^
Age (years), *n* (%)				0.66
18–24	344 (23.3%)	169 (23.7%)	175 (23.0%)	
25–34	639 (43.4%)	301 (42.2%)	338 (44.5%)	
35–44	491 (33.3%)	244 (34.2%)	247 (32.5%)	
BMI category, *n* (%)				0.24
Healthy weight	706 (47.9%)	341 (47.8%)	365 (48.0%)	
Overweight	339 (23.0%)	153 (21.4%)	186 (24.5%)	
Obese	429 (29.1%)	220 (30.8%)	209 (27.5%)	
Residence, *n* (%)				0.63
USA	1,046 (71.0%)	502 (70.3%)	544 (71.6%)	
Other	428 (29.0%)	212 (29.7%)	216 (28.4%)	
Ethnicity, *n* (%)				0.19
White	1,087 (74.1%)	518 (72.5%)	569 (75.7%)	
Other	379 (25.9%)	196 (27.5%)	183 (24.3%)	
Education, *n* (%)				0.25
High school or less	105 (7.1%)	57 (8.0%)	48 (6.3%)	
More than high school	1,369 (92.9%)	657 (92.0%)	712 (93.7%)	
Income, *n* (%)				0.88
Lower income	341 (23.1%)	161 (22.5%)	180 (23.7%)	
Middle income	573 (38.9%)	283 (39.6%)	290 (38.2%)	
Higher income	465 (31.5%)	222 (31.1%)	243 (32.0%)	
Don’t know	95 (6.4%)	48 (6.7%)	47 (6.2%)	
Stress, *n* (%)				0.64
Low	157 (10.7%)	79 (11.1%)	78 (10.3%)	
Average	1,072 (72.7%)	525 (73.5%)	547 (72.0%)	
High	185 (12.6%)	82 (11.5%)	103 (13.6%)	
No answer	60 (4.1%)	28 (3.9%)	32 (4.2%)	
Medical condition(s), *n* (%)				1.00
Has medical condition(s)	435 (29.5%)	211 (29.6%)	224 (29.5%)	
No medical condition	1,039 (70.5%)	503 (70.4%)	536 (70.5%)	
Smoking status, *n* (%)				0.16
Never	1,034 (70.1%)	514 (72.0%)	520 (68.4%)	
Rarely	273 (18.5%)	118 (16.5%)	155 (20.4%)	
Often	167 (11.3%)	82 (11.5%)	85 (11.2%)	
Copper IUD, *n* (%)				0.82
Copper IUD fitted	142 (9.6%)	67 (9.4%)	75 (9.9%)	
Not fitted	1,332 (90.4%)	647 (90.6%)	685 (90.1%)	
Vaccine doses, *n* (%)				0.37
Yes, one shot	33 (2.2%)	20 (2.8%)	13 (1.7%)	
Yes, three shots	644 (43.7%)	309 (43.3%)	335 (44.1%)	
Yes, two shots	797 (54.1%)	385 (53.9%)	412 (54.2%)	
First vaccine type, *n* (%)				0.34
mRNA	1,283 (87.4%)	628 (88.3%)	655 (86.5%)	
Other	185 (12.6%)	83 (11.7%)	102 (13.5%)	
Survey time (seconds), Median (Q1, Q3)	252 (216, 280)	252 (214, 279)	252 (217, 282)	0.65
Cycle length (days), Median (Q1, Q3)	28 (27, 31)	28 (27, 30)	29 (27, 31)	**<0.001**
Menses length (days), Median (Q1, Q3)	4.00 (3.00, 5.00)	4.00 (3.00, 5.00)	4.00 (3.00, 5.00)	0.98

Demographic, health, lifestyle, and vaccination characteristics of participants (*n* = 1474) classified by menstrual cycle phase at the time of first COVID-19 vaccination. Continuous variables are presented as median with interquartile range (IQR); categorical variables as count and percentage. Between-group comparisons were performed using Wilcoxon rank-sum tests for continuous variables and chi-square or Fisher’s exact tests for categorical variables. No significant differences were observed between the follicular (*n* = 760) and luteal (*n* = 714) groups, except for a one-day difference in median cycle length. Significant values (*p* < 0.05) are in bold.

*BMI* body mass index, *IQR* interquartile range, *mRNA* messenger ribonucleic acid.

^a^*n* (%); Median (Q1, Q3) for continuous variables

^b^Wilcoxon rank-sum test; Chi-square or Fisher’s exact test

### Menstrual cycle phase at vaccination and side effect outcomes

Following the first COVID-19 vaccination, a majority (73.2%) of participants self-reported they experienced any side effects, 75.9% following follicular-phase vaccination and 70.3% following luteal-phase vaccination. Most respondents (overall = 48.2%; follicular = 50.8%, luteal = 45.4%) self-reported weak side effects, and the majority (74.9%), self-reported 3 or fewer side effects (Table 2). The most commonly self-reported side effects were “pain at injection site” (61.5%), “fatigue” (51.0%), and “aches/pains” (35.7%), aligning with previous COVID-19 vaccine studies^60,61^.

**Table 2:** Side effect outcomes: presence, severity, number and type, by menstrual cycle phase at first COVID-19 vaccination

Characteristic	Overall *N* = 1474^a^	Follicular *N* = 760^a^	Luteal *N* = 714^a^
Presence of side effects, *n* (%)	1079 (73.2%)	577 (75.9%)	502 (70.3%)
Side effect severity, *n* (%)			
None	395 (26.8%)	183 (24.1%)	212 (29.7%)
Weak	710 (48.2%)	386 (50.8%)	324 (45.4%)
Moderate	234 (15.9%)	111 (14.6%)	123 (17.2%)
Severe	135 (9.2%)	80 (10.5%)	55 (7.7%)
Number of side effects, *n* (%)			
0	396 (26.9%)	184 (24.2%)	212 (29.7%)
1	201 (13.6%)	111 (14.6%)	90 (12.6%)
2	269 (18.2%)	141 (18.6%)	128 (17.9%)
3	238 (16.1%)	121 (15.9%)	117 (16.4%)
4+	370 (25.1%)	203 (26.7%)	167 (23.4%)
Injection site pain, *n* (%)	907 (61.5%)	478 (62.9%)	429 (60.1%)
Fatigue, *n* (%)	752 (51.0%)	412 (54.2%)	340 (47.6%)
Aches and pains, *n* (%)	526 (35.7%)	286 (37.6%)	240 (33.6%)
Headache, *n* (%)	407 (27.6%)	218 (28.7%)	189 (26.5%)
Chills/fever, *n* (%)	306 (20.8%)	165 (21.7%)	141 (19.7%)
Change in period, *n* (%)	168 (11.4%)	79 (10.4%)	89 (12.5%)
Other, *n* (%)	62 (4.2%)	37 (4.9%)	25 (3.5%)
Sore throat, *n* (%)	45 (3.1%)	22 (2.9%)	23 (3.2%)
Diarrhoea, *n* (%)	28 (1.9%)	16 (2.1%)	12 (1.7%)
Rash, *n* (%)	24 (1.6%)	8 (1.1%)	16 (2.2%)
Chest pain/pressure, *n* (%)	15 (1.0%)	10 (1.3%)	5 (0.7%)
Dry cough, *n* (%)	14 (0.9%)	7 (0.9%)	7 (1.0%)
Difficulty breathing, *n* (%)	14 (0.9%)	8 (1.1%)	6 (0.8%)
Conjunctivitis, *n* (%)	2 (0.1%)	0 (0.0%)	2 (0.3%)
Loss of speech/movement, *n* (%)	1 (0.1%)	1 (0.1%)	0 (0.0%)
Loss of taste, *n* (%)	1 (0.1%)	1 (0.1%)	0 (0.0%)

Self-reported side effect outcomes following first COVID-19 vaccination, stratified by menstrual cycle phase. Side effect presence (binary), severity (ordinal: none, weak, moderate, severe), number (count, 0–16), and type are presented for the full sample (*n* = 1474) and by follicular (*n* = 760) and luteal (*n* = 714) phase groups. Between-group comparisons used chi-square tests for categorical variables and Wilcoxon rank-sum tests for continuous variables.

^a^*n* (%); Median (Q1, Q3) for continuous variables

To account for multiple comparisons across our three primary models (binary, ordinal, and negative binomial), we applied a Bonferroni correction. Adjusted *p*-values were calculated by multiplying the raw *p*-values by three. Significance was defined as adjusted *p* < 0.05, which corresponds to the 98.3% confidence intervals reported for all estimates. Raw *p*-values are also reported for transparency.

**Fig. 2:**
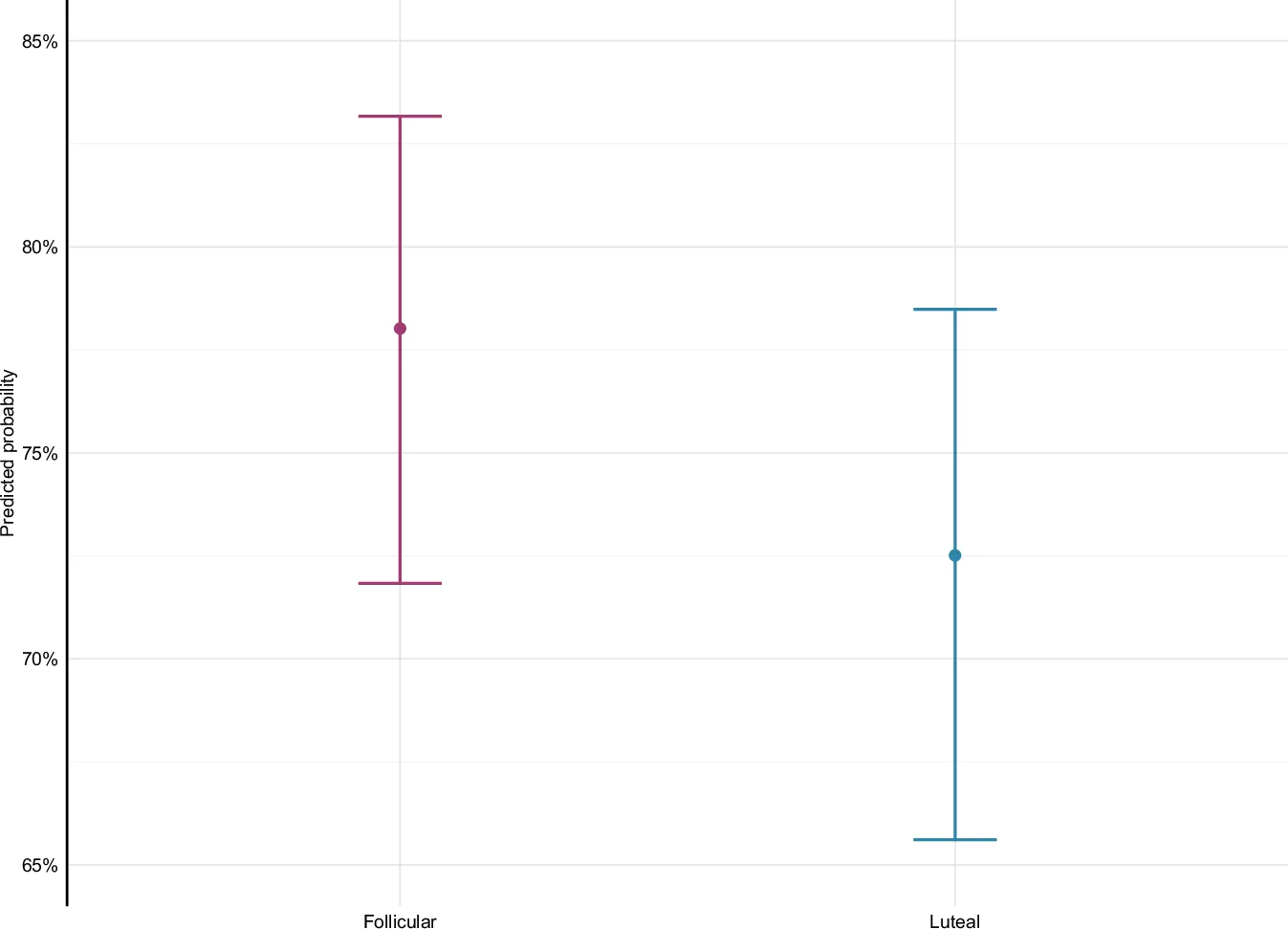
Predicted probabilities of any reported side effects by cycle phase at first vaccination, adjusted for confounders. Error bars represent 98.3% confidence intervals (CI). Follicular phase: 78% (98.3% CI: 73%, 82%); Luteal phase: 73% (98.3% CI: 67%, 77%). Predicted probabilities are adjusted for age group (25–34 years), Body mass index (BMI) category (healthy weight), pre-existing medical conditions (none), and smoking status (never).

Vaccination during the follicular phase was associated with 35% higher odds of self-reporting side effects compared to luteal-phase vaccination in a binary logistic regression model adjusted for age, BMI, smoking status, and pre-existing medical conditions (OR: 1.35, 98.3% CI: 1.01–1.79). The only other significant predictor of self-reporting side effects was age, with the youngest participants (aged 18–24) less likely to self-report side effects than those aged 25–34 (OR: 0.67, 98.3% CI: 0.47–0.96; Fig. 2; Table 3A). The association between follicular-phase vaccination and higher odds of self-reporting side effects persisted in sensitivity analyses restricted to participants without factors that might confound the association (no medical conditions, never-smokers, non-obese, no copper IUD, post-secondary education), with effect sizes ranging from OR = 1.29 to 1.54 (all *p* ≤ 0.05; Supplementary Table 1).

**Table 3:** Self-reported side effect presence, severity and number by menstrual cycle phase at first COVID-19 vaccination: Binary logistic, ordinal logistic and negative binomial regression results

	a) Presence				b) Severity				c) Number			
Characteristic^a^	OR^a^	98.3% CI^a^	*p*-value (raw)^a^	*p*-value (adj.)^a^	OR^a^	98.3% CI^a^	*p*-value (raw)^a^	*p*-value (adj.)^a^	IRR^a^	98.3% CI^1^	p-value (raw)^1^	p-value (adj.)^1^
Cycle phase (14-day luteal)												
Luteal	—	—			—	—			—	—		
Follicular	**1.35**	**1.01, 1.79**	**0.013**	**0.038**	1.21	0.95, 1.52	0.056	0.2	1.08	0.96, 1.22	0.11	0.3
Age group (per 10 years)												
25-34	—	—			—	—			—	—		
18-24	**0.67**	**0.47, 0.96**	**0.007**	**0.021**	0.76	0.56, 1.03	0.029	0.086	0.90	0.78, 1.05	0.12	0.4
35-44	1.19	0.85, 1.68	0.2	0.7	1.15	0.88, 1.51	0.2	0.6	0.97	0.85, 1.11	0.6	>0.9
BMI category												
Healthy weight	—	—			—	—			—	—		
Obese	0.94	0.67, 1.32	0.7	>0.9	0.97	0.73, 1.28	0.8	>0.9	1.02	0.89, 1.17	0.8	>0.9
Overweight	1.04	0.73, 1.51	0.8	>0.9	1.09	0.81, 1.46	0.5	>0.9	1.05	0.90, 1.22	0.4	>0.9
Medical condition(s): Yes (vs No)												
No medical condition	—	—			—	—			—	—		
Has medical condition(s)	0.88	0.63, 1.22	0.3	>0.9	0.99	0.76, 1.30	>0.9	>0.9	1.00	0.88, 1.15	>0.9	>0.9
Smoking status												
Never	—	—			—	—			—	—		
Often	1.28	0.81, 2.11	0.2	0.6	1.22	0.84, 1.77	0.2	0.6	1.14	0.95, 1.37	0.083	0.2
Rarely	0.78	0.55, 1.13	0.10	0.3	0.81	0.60, 1.11	0.11	0.3	0.92	0.78, 1.07	0.2	0.6

a) Binary logistic regression model; ¹OR = Odds Ratio. N = ; AIC = 1707.7; BIC = 1755.4; Deviance = 1689.7; Null deviance = 1713.5; Pseudo R² = 0.014; Log-likelihood = -844.9

b) Ordinal regression model; ²OR = Odds Ratio. N = ; AIC = 3587.1; BIC = 3645.4; Residual Deviance = 3565.145; Log-Likelihood = -1782.573; df = 4411

c) Negative binomial regression model; ³IRR = Incidence Rate Ratio. N = ; AIC = 5779.1; BIC = 5832.1; Deviance = 1775.6; Null deviance = 1787; Pseudo R² = 0.006; Log-likelihood = -2879.6; θ (dispersion) = 2.326; SE(θ) = 0.211; Dispersion ratio = 0.881

Regression results for the association between menstrual cycle phase at first COVID-19 vaccination and three self-reported side effect outcomes (*n* = 1474). All models are adjusted for age, BMI, smoking status, and pre-existing medical conditions. A Bonferroni correction was applied across the three models; significance is defined as adjusted *p* < 0.05, corresponding to the 98.3% confidence intervals reported. a) Binary logistic regression for presence of any side effects. b) Ordinal logistic regression for side effect severity. c) Negative binomial regression for number of side effects reported.

*OR* odds ratio, *IRR* incidence rate ratio, *CI* confidence interval, *BMI* body mass index, *AIC* Akaike information criterion, *BIC* Bayesian information criterion, *SE* standard error.

^a^Results are bolded where the Bonferroni adjusted *p*-value (*p*-adj) < 0.05. Confidence Intervals are calculated at the 98.3% level.

We conducted sensitivity analyses to eliminate the possibility of self-reported vaccine side effects being driven by menstrual or perimenstrual symptoms. The association was confirmed after excluding vaccination in the perimenstrual period (3 days before through 2 days after menstrual onset^62^; *n* = 337; OR: 1.42, 98.3% CI: 1.05–1.92). After excluding participants vaccinated during menstruation (*n* = 214), the effect size remained stable but did not meet the adjusted significance threshold (OR: 1.33, 98.3% CI: 1.00–1.82). Similarly, when excluding the periovulatory window (days −15 to -12 before menstruation^62^; *n* = 196) the effect size remained but the association failed to reach the adjusted significance level (OR: 1.28, 98.3% CI: 0.94–1.75).

To determine if the cycle-phase association with self-reporting any side-effects reflected systemic or localised side effects, we excluded participants who only reported pain at the injection site (the only localised side effect; *n* = 153; Table 2). The effect size remained stable but did not meet the adjusted significance threshold (OR: 1.33, 98.3% CI: 0.99–1.77).

Finally, we found that younger participants (18–24 years) showed a stronger follicular-phase association (OR: 2.18, 95% CI: 1.24–3.84) than the overall sample (Supplementary Table 1). This was driven by lower side effect self-reporting during the luteal phase in younger participants, while their follicular-phase reporting matched the full sample (Supplementary Table 2).

To investigate whether the overall lower reporting rates in younger participants (18–24 years) were driven by underlying socio-demographic or health-related confounders, we examined how participant characteristics varied across age categories (Supplementary Table 3). Time taken to complete the survey did not differ between the age groups, suggesting lower side effect reporting is not a result of incomplete responses by the younger group. The 18–24 cohort did differ from older participants in BMI, income, education, pre-existing health conditions, cycle*-*length and parity, but did not differ in smoking behaviour or type of vaccine received (Supplementary Table 3). To determine if these differences between age groups could explain lower side-effect reporting we ran an expanded multivariable model incorporating BMI, income, education, ethnicity, parity, smoking and vaccine type. Young age was significantly associated with lower odds of self-reporting side effects compared to the 25–34 age group (0.65, 98.3%CI 0.48–0.89) while the socio-demographic and health variables showed no significant association (*p* > 0.05 for all), although recipients of non-mRNA vaccines had significantly higher odds of reporting side effects compared to mRNA recipients (OR: 2.32, 98.3% CI: 1.53–3.64, full model results in Supplementary Table 4*)*. In the context that none of the factors which differed between age groups associated with odds of self-reporting side effects, the observation that this difference is driven by lower luteal-phase reporting, points to a physiological rather than a socio-demographic explanation.

**Fig. 3:**
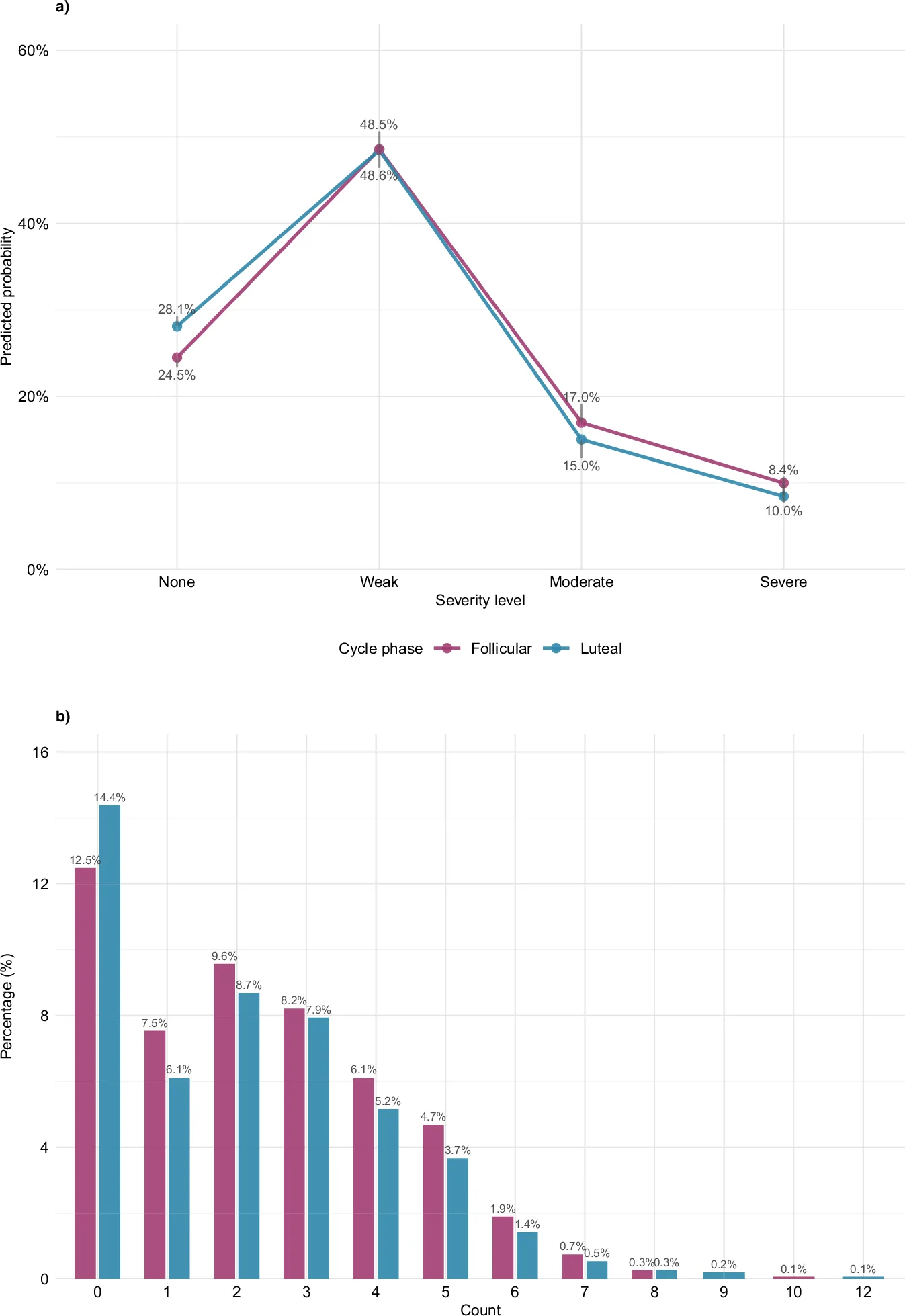
Side effect severity and number by menstrual cycle phase at first COVID-19 vaccination. Side effect severity (**a**) and number (**b**) by menstrual cycle phase at first COVID-19 vaccination. **a** Predicted probability of reporting side effects by severity level (none, mild, moderate, severe) for follicular (blue) and luteal (purple)-phase vaccination. Probabilities are adjusted for age, Body mass index (BMI), medical conditions and smoking status. **b** Distribution of number of side effects reported (0–12) by menstrual cycle phase at first COVID-19 vaccination. Most participants reported 0–3 side effects regardless of cycle phase, with frequency decreasing as number of reported side effects increases.

Individuals vaccinated in the follicular phase did not have higher odds of self-reporting more severe side effects compared to those vaccinated in the luteal phase (OR: 1.21, 98.3% CI: 0.95-1.52, Fig. 3a, Table 3b) in an ordinal logistic regression model adjusted for age, BMI, smoking status, and pre-existing medical conditions. This finding was consistent across sensitivity analyses (Supplementary Table 5).

Follicular-phase vaccination was not associated with the number of reported side effects (IRR: 1.03, 98.3% CI: 0.96–1.22; Table 3C, Fig. 3b) in a negative binomial model adjusted for age, BMI, smoking status, and pre-existing medical conditions, a finding consistent across sensitivity analyses (Supplementary Table 6).

### Menstrual cycle-phase at vaccination and COVID-19 infection

**Fig. 4:**
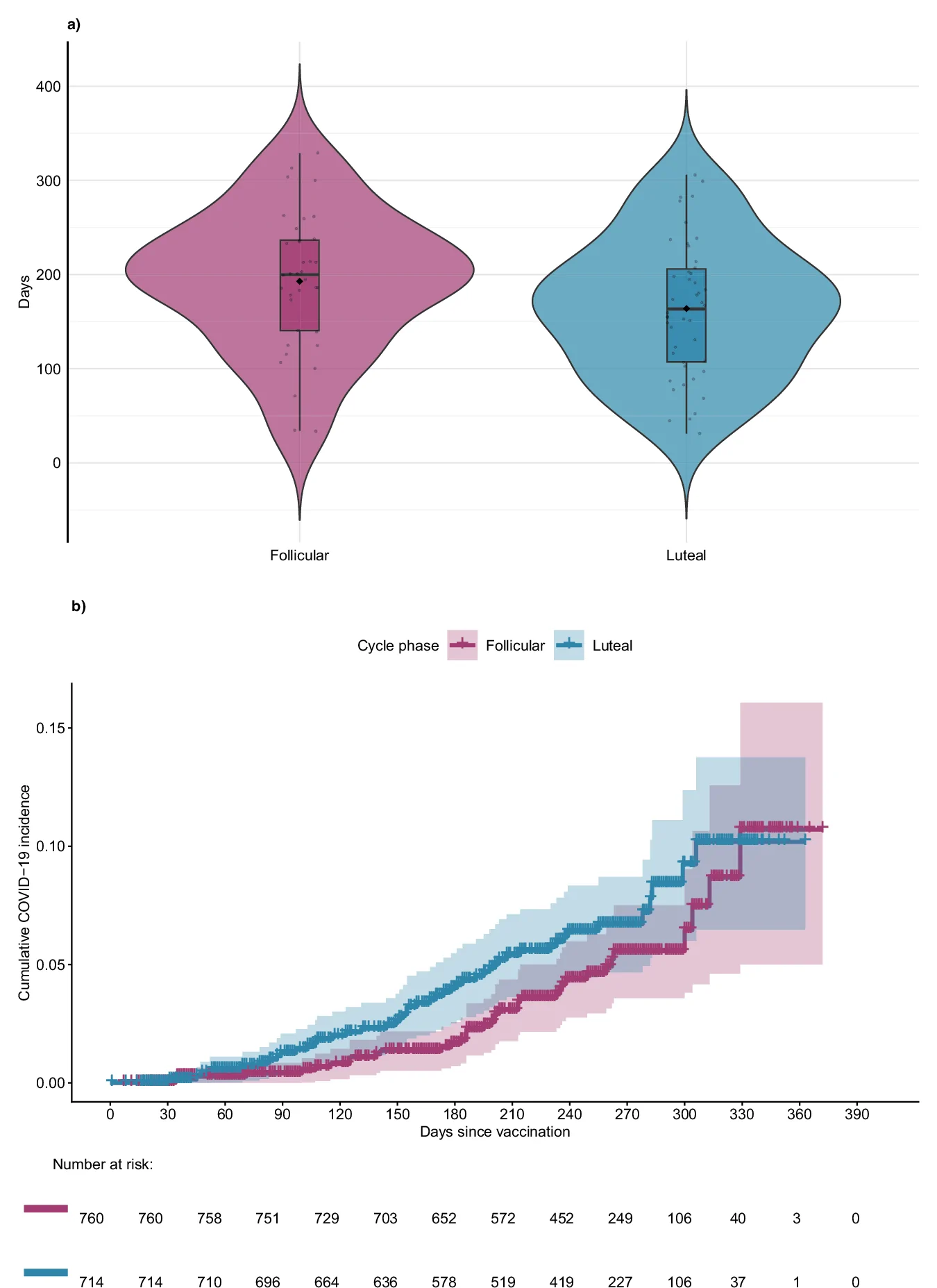
COVID-19 time to infection and cumulative incidence by menstrual cycle phase at first COVID-19 vaccination. COVID-19 Time to infection (**a**) and cumulative infection incidence (**b**) by cycle phase (follicular/luteal) at first COVID-19 vaccination. **a** Time to COVID-19 infection by menstrual cycle phase (follicular/luteal) at first vaccination. Violin plots with embedded box plots show median time to infection was significantly longer for follicular phase (200 days, interquartile range (IQR): 140–237) compared to luteal phase (164 days, IQR: 107–207; *p* = 0.05). **b** Cumulative COVID-19 incidence by cycle phase (follicular/luteal) at first COVID-19 vaccination. Cumulative incidence curves show overall infection rates were similar (4.7% vs 6.4%, *p* = 0.19), but luteal-phase infections occurred earlier.

To test the hypothesis that menstrual cycle phase at vaccination influences subsequent COVID-19 infection risk, we analysed infection outcomes during the observation period (67 to 372 days post-vaccination). Of the 1474 participants, 82 (5.6%) self-reported a break-through COVID-19 infection. Participants vaccinated during the follicular phase exhibited a longer median time to infection (200 days; IQR: 140–237) compared to those vaccinated in the luteal phase (164 days; IQR: 107–207; Wilcoxon rank-sum test, *p* = 0.05, Table 4, Fig. 4a and 4b).

**Table 4:** COVID-19 outcomes post vaccination by menstrual cycle phase at vaccination

Outcome	Overall *N* = 1474^1^	Follicular *N* = 760^a^	Luteal *N* = 714^a^	*p*-value^b^
**COVID-19 infection**	82 (5.6%)	36 (4.7%)	46 (6.4%)	0.19
**Time to COVID-19 (days)**	184 (123, 233)	200 (140, 237)	164 (107, 207)	**0.05**
**COVID-19 symptom severity**				0.9
I did not experience symptoms	1 (1.2%)	0 (0.0%)	1 (2.2%)	
I had some mild cold symptoms, but they didn’t interfere with my activities	18 (22.0%)	9 (25.0%)	9 (19.6%)	
I had moderate symptoms that interfered with my activities	29 (35.4%)	13 (36.1%)	16 (34.8%)	
I had moderate symptoms that prevented me from my activities	32 (39.0%)	13 (36.1%)	19 (41.3%)	
I had severe symptoms, and I had to get emergency care, but I did not need to stay in the hospital	2 (2.4%)	1 (2.8%)	1 (2.2%)	
I had severe symptoms, and I needed oxygen therapy in the hospital, but I did not need a ventilator	0 (0.0%)	0 (0.0%)	0 (0.0%)	

^a^COVID-19 infection shown as *n* (%); Time to COVID-19 shown as median (IQR) for infected participants

^b^Pearson’s Chi-squared test; Wilcoxon rank sum test

Self-reported COVID-19 infection and time to infection following first vaccination, stratified by menstrual cycle phase at vaccination. Of 1474 participants, 82 (5.6%) reported a breakthrough infection during the observation period (67–372 days post-vaccination). Infection frequency is presented as count and percentage; time to infection is presented as median with interquartile range (IQR) for infected participants only. Between-group comparisons used Pearson’s chi-squared test for infection frequency and a Wilcoxon rank-sum test for time to infection.

We ran a Cox proportional hazards model to account for censoring and potential confounders; however, the limited number of infection events (*n* = 82) meant the model had only 29% power at α = 0.05 (model results in Supplementary Table 7 for information). Consequently, we cannot draw definitive conclusions regarding differential infection risk. The Wilcoxon rank-sum results should be interpreted as descriptive and hypothesis-generating rather than inferential

### **Alternative cycle-**phase classification methods

Our method for determining cycle-phase at vaccination relied on calendar-based estimation; classifying the last 14 days of the cycle as the luteal phase and the remainder as the follicular phase. However, the 14-day luteal phase is not universal: a study of over 600,000 cycles from the Apple Women’s Health Study found the average luteal phase to be 12.4 days, and that it varied by age and cycle length^63^. We therefore tested the associations using alternative luteal-phase lengths (12-day, 13-day, age-adjusted, and cycle-length-adjusted classifications based on the Apple Women’s Health Study’s findings). Regardless of classification method, the associations between cycle phase at vaccination and side effect outcomes persisted in the same direction. The 14-day luteal classification consistently provided the best or equivalent model fit across all outcomes and was therefore selected as the primary analysis approach (Supplementary Table 8).

While binary classification of the menstrual cycle into two distinct phases permits clear, interpretable analysis, it fails to account for hormonal fluctuations within the follicular and luteal phases^15^. To determine whether the probability of self-reporting side effects varied within the phases, we conducted an exploratory analysis using Phase-Aligned Cycle Time Scaling (PACTS) via the *menstrualcycleR* package^64^. This method standardises diverse cycle lengths to a linear 0–1 scale, anchoring the onset of the current menstruation at 0 and the subsequent menstruation onset at 1. Mirroring our binary classification, ovulation was imputed at 15 days prior to the subsequent menses and anchored at the 0.5 midpoint. This approach allows us to detect variability in odds of self-reporting side effects across the whole cycle, that a binary model would otherwise aggregate, while ensuring that biological transitions (ovulation and menstruation) remain aligned across participants regardless of total cycle duration.

**Fig. 5:**
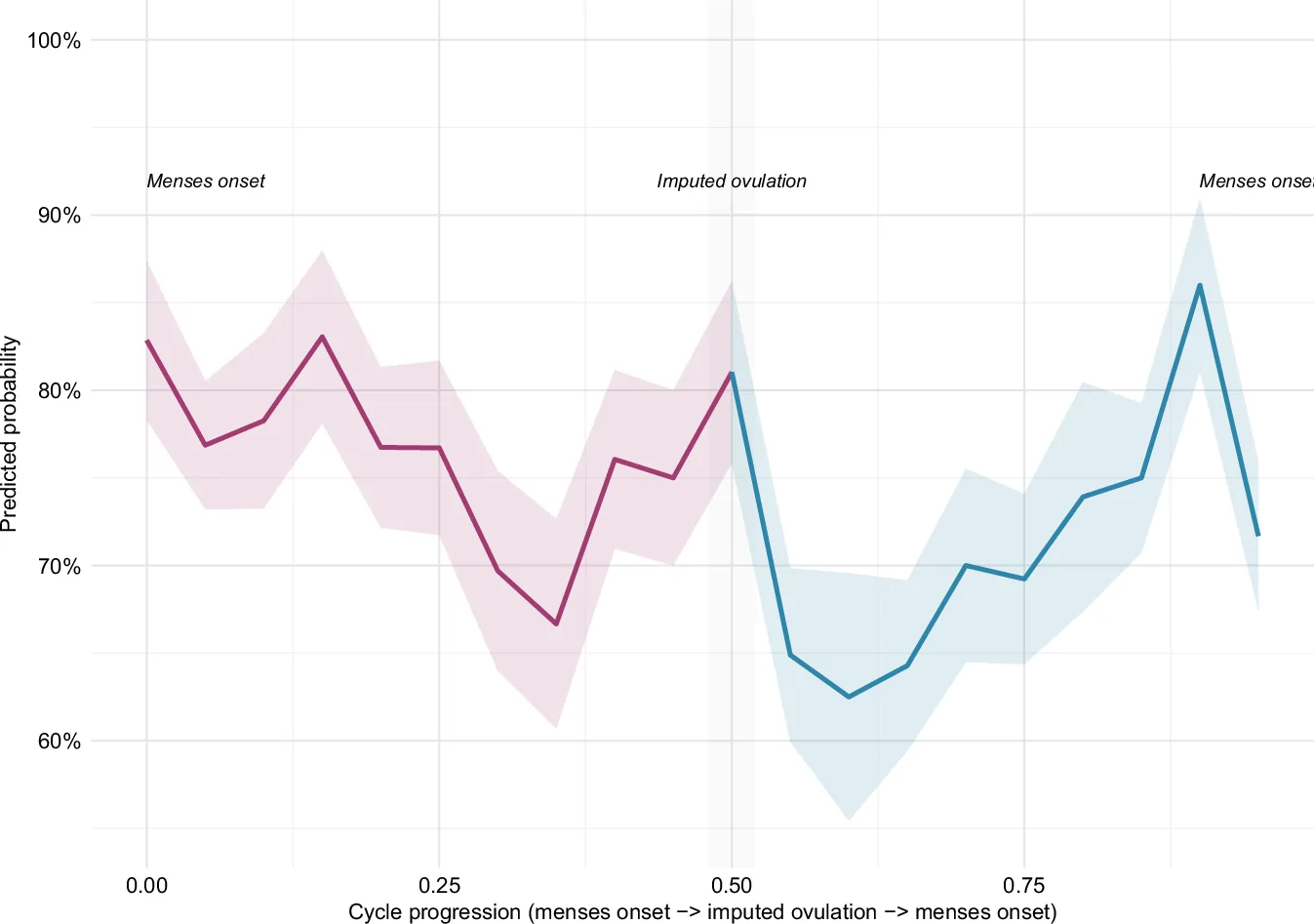
Probability of self-reported side effects throughout the menstrual cycle. Plot shows the probability of self-reporting any side effects following first COVID-19 vaccination across a standardised menstrual cycle. Menses onset is anchored at 0 and 1, with ovulation at 0.50. Points are plotted at 0.05 increments. Shaded areas represent ±1 Standard Error (SE).

Visualising predicted probabilities in 0.05 increments of cycle progression revealed notable intra-phase variability and a distinct bimodal pattern consistent with our primary categorical findings (Fig. 5; Supplementary Table 9). The cycle begins at menses onset (0.00) with a high probability of self-reporting side effects (83.3%), followed by an immediate dip to 69.9% (0.05 progression through the cycle) before rising back to 83.1% during the mid-follicular phase (0.15 progression). A secondary decline occurs as the follicular phase progresses, reaching a follicular-phase low of 66.7% at 0.35 progression. Probability then rises steadily through the periovulatory window, peaking at 81.0% at the estimated day of ovulation (0.50), mirroring the physiological oestrogen surge. Immediately following ovulation, the lowest probabilities of the entire cycle are observed during the early luteal phase, reaching a nadir of 62.5% at 0.60 progression, as progesterone begins to rise. The latter half of the cycle is characterised by a gradual increase during the mid-to-late luteal phase, culminating in a late-cycle peak of 86.0% at 0.90 progression, before a final decline immediately prior to the next menses onset. The pattern is consistent with our primary finding of greater odds of self-reported side effects following follicular-phase vaccination, while additionally capturing perimenstrual elevations and the early luteal nadir that fixed-phase classification cannot detect.

To further investigate the 18–24 age group—which showed a stronger association between follicular phase vaccination and any self-reported side effects in the primary analysis (Supplementary Table 1)— the continuous PACTS analysis was repeated exclusively for this cohort. While the resulting trend mirrored the pattern of the full model (see Supplementary Fig. 1), the intra-phase fluctuations were more pronounced. Notably, the 18–24 cohort exhibited a deeper early luteal nadir, with the probability of self-reporting side effects dropping to 38.9% (0.70 progression), compared to 62.5% in the full model (Supplementary Table 10).

## Discussion

Despite 1.8 billion people globally experiencing menstrual cycles each month (https://www.unicef.org/wash/menstrual-hygiene), historical biases^65,66^, persistent social stigmas and taboos^67,68^, and practical challenges in data collection^62,69^, mean the menstrual cycle’s impact on non-reproductive health remains understudied. Using prospectively collected menstrual cycle data matched to self-reported survey data on COVID-19 vaccine experiences and infection dates, we found that follicular-phase vaccination was associated with a 35% increased likelihood of self-reporting side effects compared to luteal-phase vaccination. We also observed a 5-week longer self-reported median time to breakthrough infection in women who received a follicular-phase vaccination; however, this finding was insufficiently powered for formal inference and remains exploratory. Together these findings represent preliminary evidence that menstrual cycle phase at the time of vaccination may influence vaccine reactogenicity and warrants further investigation regarding protective efficacy.

The internal validity of these findings is supported by the prospective nature of the cycle data, which minimises the recall bias inherent in retrospective surveys. Focusing on the first vaccine dose further avoids confounding from cumulative or mixed vaccine exposures. DAG - informed covariate selection, and the consistency of the association across multiple sensitivity analyses, including never-smokers, non-obese participants, non-copper IUD users, and those without pre-existing conditions (Supplementary Table 1), further support a true association between cycle-phase at vaccination and odds of self-reporting side effects. Additionally, we excluded hormonal contraceptive users, participants aged 45 +, and those reporting irregular cycles (Fig. 1). The observed cycle-related differences are therefore unlikely to reflect hormonal contraceptive symptoms^70^, low-grade inflammation linked to obesity^71,72^ perimenopause symptoms^73^, copper IUD symptoms^74,75^, smoking^76–78^ or underlying medical conditions^79^ rather than cyclical hormone changes. However, as a self-selected sample of app users, participants differ from the general population in health behaviours, digital literacy, and socioeconomic status, being predominantly white, educated and affluent USA residents (Table 1). Furthermore, while the prospective cycle tracking reduces specific dating errors, survey data regarding the vaccine experience were collated a median of 252 days (IQR 216–280) after the first dose. While recall bias regarding side effects is a possibility over this duration, the time from vaccination to survey did not differ significantly between the follicular and luteal groups (Table 1), suggesting that any recall attenuation was likely balanced across the cohorts.

The higher odds of any reported side effects following follicular-phase vaccination align with the pro-inflammatory nature of menstruation as well as rising estrogen leading up to ovulation which could promote cytokine production^17,32,35,80^. In contrast, progesterone’s immunosuppressive effects^81–83^ might explain the lower overall odds of self-reported side effect in the luteal phase^15,84^, despite the inflammatory nature of the premenstrual period. Our exploratory PACTS analysis using a standardised, continuous menstrual cycle variable (Supplementary Table 9) provides high-resolution support for this interpretation. The visual plot (Fig. 5) reveals a steady rise in the probability of self-reported side effects during the late follicular phase as ovulation approaches—mirroring the physiological estrogen peak—and a notable low in the early luteal phase, coinciding with the estrogen drop and progesterone rise. Vaccine reactogenicity is a plausible proxy for innate immune activation^85^ and these findings suggest that the follicular phase may be a window of heightened immunological responsiveness. This interpretation aligns with evidence that symptoms of epilepsy, asthma, rheumatoid arthritis, irritable bowel syndrome, and diabetes fluctuate across the menstrual cycle^86^.

However, the nuance of this association warrants cautious interpretation regarding the subjective nature of self-reported side effects. When restricted to participants who self-reported systemic side effects (excluding ‘*Injection Site* *Pain*’) the observed association lost statistical significance (Supplementary Table 1). This could indicate the observed association is driven by cyclical changes in pain perception rather than immune response. Increased pain sensitivity has been linked to high levels of estradiol^87^, though more consistently with fluctuating levels^87–89^, such as those that characterise the follicular phase. Rising progesterone post-ovulation may result in ‘Luteal Analgesia^90^” where side effects, including injection site pain, are less noticeable owing to the dampening effects of progesterone and its metabolite, allopregnanolone, on pain signalling^90–92^. The self-reported nature of the data also introduces the potential for participants to misattribute other standard menstruation symptoms (fatigue, headache, breast tenderness and cramping^93^) to the vaccine, inflating follicular-phase reporting, which could explain why the association lost significance when participants vaccinated in their bleed phase were excluded. Similarly, periovulatory symptoms such as migraines and ovulation pain, may also have been misattributed, explaining the loss of significance in the periovulatory sensitivity analysis.

Nevertheless, the persistence of the association after excluding perimenstruation - the five-day window spanning the final three days of the cycle and the first two of the subsequent cycle^62^- is particularly noteworthy. This period is hormonally distinct, marked by low estrogen, low progesterone and high systemic inflammation^44^, and typically represents the most symptom heavy time of the cycle^62,94^. By removing participants vaccinated in this window, the analysis retains a sample least likely to confuse baseline menstrual symptoms with vaccine side effects. Further, it facilitates a clearer comparison of those vaccinated later in the follicular phase (rising estrogen, low progesterone) and the earlier luteal phase (falling estrogen, rising progesterone). The continued significance of the association in this context provides support for a genuine hormonal influence on reactogenicity. This is further underscored by our exploratory PACTS analysis, which revealed that side effect reporting rose immediately before and after menses, highlighting the limitations of a binary cycle variable in capturing non-static hormonal environments^95^ (Fig. 5, Supplementary Table 9).

While these findings point to a biological reality, they must be interpreted alongside the inherent challenges of estimating hormonal phases without direct biomarkers^62^. Cycle phase was estimated from tracking data; ovulation was inferred rather than measured, and hormone levels were not available. It’s possible, for instance, the periovulatory sensitivity analysis incorrectly excluded people entirely before or after ovulation. Ovulation may be absent in some cycles, resulting in different hormone profiles from the one assumed, and differing corresponding immune responses^84^. Irregular cycles can indicate underlying health conditions which disrupt ovulation, so excluding participants who did not meet the FIGO criteria for regular cycles^59^ is a buffer against this. The assumed 14-day luteal phase classification proved the best fit to the data, but such a rigid metric is not representative of real-world variation^63^. This assumption creates a structural imbalance: participants with longer cycles are classified as follicular phase for a greater proportion of their cycle, meaning more vaccination dates fall within the follicular-phase classification than the luteal-phase (Table 1). This could introduce confounding if cycle length itself relates to immune response, or if individuals with systematically longer cycles differ in unmeasured ways not captured in our DAGs (Supplementary Fig. 2 and Supplementary Fig. 3). Future studies using biological confirmation of ovulation and cycle phase, combined with direct hormone measurements, would help disentangle these potential sources of confounding.

The potential for incorrect cycle-phase classification could be particularly relevant when examining the youngest cohort (ages 18–24), who exhibited both the longest median cycle length and the strongest association between vaccination cycle-phase and self-reported side effects (Supplementary Table 3). Because this group had a median cycle of 30 days (IQR-28–32) compared to 28 days (IQR 26–29) in the 35–44 group and 29 (IQR 27–31) in the 25–34 group, younger participants were disproportionately classified as follicular-phase, increasing their susceptibility to the misclassification bias described above.

The lower overall side-effect reporting in this group is less easily explained by methodology, however (Table 3). It is unlikely the younger group under-reported side effects as survey time did not differ between age groups (Supplementary Table 3), and previous studies have not found youth to be associated with lower side-effect reporting following COVID-19 vaccination, even sometimes finding the reverse, particularly when using web-based collection methods^95^. The 18–24 age group differed significantly from the older groups in baseline health, parity, and socio-economic status (Supplementary Table 3*)*, but our exploratory multivariable regression analysis found that none of these covariates associated with odds of self-reporting side effects (Supplementary Table 4*)*. Crucially, the raw data reveal that the lower self-reporting of side effects in the 18–24 age group was driven specifically by lower rates in the luteal phase, rather than uniform reduction across the cycle (Supplementary Fig. 1). This raises the possibility that luteal-phase immunosuppression may be more pronounced in younger women—perhaps reflecting stronger or more consistent hormonal cycling—though this remains speculative. Directly testing this would require hormonal biomarker data.

Our study did not find a significant reduction in infection risk following follicular-phase vaccination in the adjusted Cox proportional hazards model. This analysis was underpowered, with just 29% power to detect the observed hazard ratio of 0.73 (Supplementary Table 7*)*. Although the unadjusted bivariate analysis showed a 5-week longer time to infection following follicular-phase vaccination (Table 4), the limited number of infection events (*n* = 82) and the need for censoring precluded confirmation in a multivariable model. Furthermore, COVID-19 infection was self-reported and limited to events between vaccination and survey completion (a period ranging from 65–372 days), introducing variability in exposure opportunity and potential recall bias. Given that the ±15-day margin of error associated with mid-month imputation is nearly half the observed 35-day difference between groups, this descriptive finding should be interpreted with considerable caution and cannot be considered reliable without replication using prospectively recorded infection dates.

Nevertheless, time to infection remains a valuable metric for assessing real-world COVID-19 vaccines protection profiles^96,97^, and the observed pattern in the bivariate analysis is consistent with established immunological mechanisms. Estrogen levels are associated with enhanced B cell activity and T follicular helper cell responses, both important for long-term immunity, while progesterone, can suppress these responses^32^.

The raw difference in time to infection, coupled with the increased odds of self-reported side effects, could therefore indicate coordinated adaptive immune activation during the estrogen-dominant follicular phase, while the progesterone-dominant luteal phase may favour tolerance and potentially less robust protection. However, greater vaccine reactogenicity does not necessarily indicate better protection, and previous research on COVID-19 vaccine reactogenicity and protection has been inconsistent^78,98,99^. Replication in larger cohorts with prospective biological sampling to directly measure immune responses would clarify whether cycle-phase has any clinically meaningful effect on vaccine-mediated protection.

Understanding whether follicular-phase vaccination is associated with both increased reactogenicity and enhanced protection, or increased reactogenicity alone, has important implications. Reproductive-aged women show greater vaccine hesitancy, with fear of side effects and potential long-term health implications often cited as reasons^100,101^. Clarifying any potential relationship between cycle phase at vaccination, side effects, and subsequent protection could help this population make more informed decisions. Additionally, further research into the already observed associations between vaccination timing and menstrual bleeding patterns and cycle length^8,13,14^, as well as potential influence on ovulation timing, could address concerns about menstrual changes and fertility that have been reported as barriers to uptake^2,102^. Our data had too few counts to examine menstrual changes post-vaccination formally (Supplementary Table 11*)*.

Effective vaccination requires a balance between immunogenicity. As sex hormones influence both, and fluctuate across the menstrual cycle, understanding their role is essential, yet remains understudied. Our findings offer preliminary evidence that cycle phase at vaccination may influence reactogenicity and raise the question of whether it could also affect vaccine-mediated protection, though this remains to be demonstrated. While COVID-19 vaccines have demonstrated clear effectiveness in reducing infection risk, understanding whether timing vaccination with menstrual cycle phase could optimise individual outcomes is an important avenue for future research. These findings highlight the broader importance of investigating hormonal diversity between and within women, not just between sexes, in immunological research.

## Methods

### Ethics declaration

This study was conducted in accordance with the Declaration of Helsinki and received ethical approval from the French National GDPR Committee (CNIL) and the London School of Hygiene & Tropical Medicine Observational/Interventions Research Ethics Committee (LSHTM Ethics Ref: 31539).

### Data sources and population

The “Period and the Pandemic” survey (Supplementary Fig. 4), conducted among users of the Clue app (BioWink GmbH, Berlin, Germany), collected self-reported data that were linked to participants’ prospectively logged menstrual cycle information. All data were obtained with participants’ consent, and identifying information was removed prior to data transfer and analysis.

Users aged 16 to 58 years old with a registered Clue account, who had consented to the use of their pseudonymised data for research purposes, were sent an in-app message inviting them to take part in the survey. Participants were eligible for inclusion in the analysis if they had experienced menstrual periods or withdrawal bleeds and had not been pregnant or breastfeeding since January 2020.

The survey was conducted between 29 November 2021 and 8 February 2022, and sent to registered Clue app users in the United States, United Kingdom, Canada, and Australia. It collected demographic data (age, ethnicity, residence), health markers (height, weight, pre-existing conditions, stress), lifestyle factors (smoking, contraceptive use), Covid-19 infection history (test type, test and diagnosis dates, symptoms, symptom onset date), vaccination details (type, date, number of doses—up to three), vaccine side effects (type: 16 options, and severity), and long COVID status (ongoing symptoms). Survey responses were paired, whenever possible, to longitudinal cycle data, including cycle and period start and end dates tracked from three cycles prior to the cycle containing a respondent’s first reported COVID-19 vaccine date and the end of the survey.

### Variables and data processing

Age was transformed into a categorical variable (18–24, 25–34 and 35–44) to maintain adequate numbers in each group). BMI was calculated using the standard formula and categorised as ‘underweight’, ‘healthy weight’, ‘overweight’ and ‘obese’ according to standard parameters^103^. Ethnicity was reported differently across the four countries, so responses were combined and split into seven categories (“White”, “Black”, “Hispanic”, “Asian”, “Middle Eastern”, “Multiple” and “Other”) and then collapsed into “White” and “Other”, owing to low counts in non-white groups (Supplementary Table 12). “Prefer not to answer”, was listed as “Missing” in the ethnicity variable. Residence was collapsed to “USA” and “Other”, owing to low counts in other countries. Education was grouped into “high school or below” and “more than high school”. Pre-existing medical conditions (for full list see Supplementary Table 12) were recoded to: “Has medical condition(s)”, “No medical conditions” and “prefer not to answer”. A composite smoking frequency score was calculated by numerically recoding tobacco, e-cigarette (vape), and cannabis use responses from 0 (“never or not in past 2 years”) to 4 (“several times a day”), then summing the recoded values across the three substances. The combined score was then capped at 5 and recoded into a categorical variable with three levels: “Never” (0), “Rarely” (1-3), and “Often” (4 + ).

Income was reported in six local currency categories for each country. These country-specific categories were numerically coded (1–6) to standardise them across UK, USA, Canada, and Australia, then recategorised as “Lower income*”* (1–2), “Middle income (3–4), and “Higher income” (5–6) groups; “Don’t know” responses were included as a separate category and removed for a sensitivity analysis. The administered vaccine was recoded according to its technology as “mRNA” (Pfizer-BioNTech/Moderna) or “Other” (Oxford-AstraZeneca/Johnson & Johnson/ Novovax). mRNA technologies accounted for >85% of responses (Table 1). Hormonal contraceptive users were excluded and copper IUD users retained, and a binary variable created (“Copper IUD fitted”/ “Not fitted”). Participants reported stress they felt during the pandemic on a 0–10 scale. This variable was recoded into three categories: “Low” (below median minus IQR), “High” (above median plus IQR), or “Average” (all others) based on the sample distribution and non-responses were listed as “No answer”.

Vaccine side effects were summarised using three variables: presence (binary), number (count, up to 16), and severity (ordinal). Severity was based on participant-reported interference with daily activities: “no side effects”, ”mild side effects that did not interfere with activities”, ” symptoms that interfered with activities”, “symptoms that prevented me from my activities” and ”symptoms that required emergency care or hospital visit”. These were recoded to “none”, “weak”, “moderate” and “severe”, with “severe” combining the last two levels owing to low counts.

We used time to COVID-19 infection as our primary endpoint for assessing vaccine protection. This measure is appropriate for COVID-19 vaccines given their incomplete protection profile (67–92% efficacy depending on platform^104^) and regular breakthrough infections, unlike vaccines with near-complete protection where binary success/failure outcomes are more appropriate. Time-to-infection allows detection of potential differences in the timing and durability of vaccine-induced protection development across menstrual cycle phases.

Time to infection was calculated from the date of the first vaccine as recorded by survey respondents to the earliest date given for any of COVID-19 symptom onset, COVID-19 positive test date, or COVID-19 positive diagnosis date. COVID-19 infection dates were self-reported by participants during the survey period (67–372 days post-vaccination). COVID-19 infection dates were only provided by month and year, so the 15th day of each reported month was assigned as the infection date to represent the midpoint of the likely infection window.

We matched reported first vaccine date to corresponding cycle date range and then assigned cycle phase (follicular/luteal) at vaccination. Luteal phase was defined as the 14 days preceding the onset of the next menstrual period, counting backward from the last day of the cycle (the day before menstruation onset) through to day -14. All remaining cycle days were assigned to the follicular phase^86^. Additional classifications were used in sensitivity analyses (see below).

Exclusions were applied as detailed in Fig. 1. A total of 13,261 registered Clue users who responded to the survey were matched to their prospective cycle data. We excluded unvaccinated individuals, those unable to recall or specify their vaccine date, and participants who reported a COVID-19 infection or gynaecological surgery prior to vaccination. Additional exclusions were based on demographics (did not identify as women, age 45 + ), underweight BMI (owing to low counts), hormonal contraceptive use (Copper IUD users were retained) and a response of “prefer not to answer” regarding pre-existing medical conditions. Participants who had not tracked the cycle in which their first COVID-19 vaccination occurred were also excluded. We defined regular cycles according to FIGO (International Federation of Gynaecology and Obstetrics) criteria (cycle length of 24–38 days, cycle length over three consecutive cycles not varying more than 9 days, menstruation length <9 days)^59^ and required participants to have at least three consecutive regular cycles prior to vaccination. We also excluded irregular trackers, defined as users who failed to track at least one metric other than menstruation dates in each of the three cycles preceding vaccination, to improve cycle phase accuracy given known inconsistencies in app-based tracking^105^. Following all other exclusions, one participant with missing ethnicity data was excluded to maintain complete case analysis for multivariable modelling. Participants were classified as vaccinated in the follicular phase (*n* = 760) or luteal phase (*n* = 714) based on their vaccination date within their menstrual cycle. Summary tables with un-collapsed variables are available in Supplementary Table 12.

### Statistical analysis

All statistical analyses were performed using R (version 4.2.1).

To assess whether menstrual cycle phase at the time of vaccination predicted vaccine-related side effects, three separate regression models were used. A binary logistic regression model was used to report presence of self-reported side effects, using the *glm* function in R. We modelled self-reported side effect severity using cumulative log-odds ordinal logistic regression, implemented using the vglm function from the *VGAM* package with the parallel odds assumption confirmed. The parallel odds assumption was tested using likelihood ratio tests comparing parallel and non-parallel models (*p* = 0.22), supporting the use of the proportional odds model. Number of side effects was modelled with negative binomial regression using the *glm.nb* function from the *MASS* package, following identification of overdispersion in an initial Poisson model.

To evaluate whether cycle phase predicted subsequent COVID-19 infection outcomes over the follow-up period, we conducted a Wilcoxon rank-sum test to compare time to median infection between the two cycle phases. Time to confirmed infection was then analysed using a Cox proportional hazards model (*coxph* function from the *survival* package), with time calculated from the date of first vaccination. Participants were censored at the date of survey completion if they did not report infection. Second vaccine doses were included as time-varying covariates to account for changes in immunity over time.

Covariate adjustment sets were selected using directed acyclic graphs (DAG; Supplementary Fig. 2 and Supplementary Fig. 3). All models testing side effect outcomes were adjusted for age, BMI, ethnicity, smoking status, and presence of pre-existing medical condition(s). Ethnicity was subsequently removed from the final models as its exclusion improved model fit and it not meaningfully alter results. The Cox proportional hazards model initially included the same variables plus dates of the second and third vaccines, as recommended by the DAG. However, influence diagnostics revealed the full model was unstable due to the small number of COVID-19 cases (*n* = 82) relative to the number of variables. We used stepwise selection to identify the minimum set of variables needed to control for confounding while maintaining model stability. The final Cox model included age, pre-existing conditions, and the second vaccine as a time-varying covariate. Results of the stepwise selection are provided in Supplementary Table 13.

We tested interactions with cycle phase at vaccination with model covariates age, BMI and smoking but not with ethnicity or pre-existing conditions because counts in the “Other” and “Has medical condition” groups were too small and heterogeneous to enable meaningful interpretation. Interactions with vaccine type, stress, copper IUD, education, Income and residence were also tested. Only statistically significant results are reported (Supplementary Table 14, Supplementary Table 15 and Supplementary Table 16).

We ran several sensitivity analyses. We re-ran our models with complete-case analysis: excluding participants who responded either “Don’t know” to Income or “No answer” to the stress variable. In categorical variables with a dominant category ( > 75%) we restricted the sample to that category: “White” (ethnicity), “More than high school” (education), “Not fitted” (copper IUD), “Never” (smoking), “Average” (stress) and “mRNA” (vaccine type). For BMI we re-ran the analysis excluding “Obese”. We also included sub-group analysis for the three age categories, “18–24”, “25–34” and “35–44”.

To evaluate whether concurrent menstrual cycle symptoms disproportionately influenced associations, we conducted three sensitivity analyses excluding participants vaccinated during symptom-heavy windows of the cycle: menstruation, peri-menstruation and periovulation. Menstruation was identified by matching vaccine dates to those falling between ‘period start’ and ‘period end’ dates inclusive, in participants’ cycle data. The perimenstrual period was defined as the three days before menses onset through to the second day of bleeding (days −3 to +2), capturing this hormonally distinct window^62^. To account for peak estrogen levels and ovulation-specific symptoms, we defined the periovulatory window as days −15 to −12 before the next menstruation (the periovulatory window^62^).

To assess our cycle phase classification method, we re-ran models with alternative luteal phase lengths. Although 14 days is the classical standard for calendar-based luteal phase length classification, analysis of 600,000 app-tracked cycles including ovulation, found a mean luteal phase of 12.4 days, with variation by age and cycle length^63^. We therefore tested luteal phases calculated as: (1) fixed 12 days for all participants, (2) fixed 13 days for all participants, (3) adjusted for age using mean values reported by Bull et al. (2019) and (4) adjusted for cycle length using mean values reported by Bull et al. (2019). We ran a final model excluding participants who were not classified in the same cycle phase by all these alternative definitions, to assess results in individuals with consistent phase classification across methods.

Beyond discrete binary classifications, we conducted an exploratory analysis using Phase-Aligned Cycle Time Scaling (PACTS) from the *menstrualcycleR* package^64^. This method mathematically standardises cycles of varying lengths, restricted in this study to a range of 24–38 days, onto a continuous scale. As biologically validated ovulation data were unavailable, ovulation was imputed at day −15 relative to the subsequent menses onset, assuming a 14-day luteal phase. On the standardised PACTS scale used for visualisation and modelling (Fig. 5), menses onset is represented by 0 for the current cycle and 1 for the next cycle, while the 0.50 midpoint represents the estimated day of ovulation, marking the transition from the follicular to the luteal phase.

Unlike traditional counting methods, PACTS uses a circular scaling approach that anchors to both menses and imputed ovulation. This allows for a high-resolution, continuous examination of how reported side effects change relative to the underlying hormonal trajectory, validating whether observed associations follow the expected hormonal fluctuations across the standardised cycle.

Three side effect analyses (binary logistic, ordinal, and negative binomial regression) comparing luteal vs follicular phase at vaccination were conducted in the full sample (*n* = 1474) and considered jointly for multiple testing. To account for multiple comparisons across our three primary side-effect models, we applied a Bonferroni correction. Adjusted *p*-values were calculated by multiplying the raw *p*-values by three. Significance was defined as adjusted *p* < 0.05, which corresponds to the 98.3% confidence intervals reported for all estimates. Raw *p*-values are also reported for transparency. The Cox proportional hazards model for time to COVID-19 infection was treated as a separate hypothesis test with a significance threshold of *α* = 0.05.

Post hoc power analyses indicated that for binary logistic and ordinal regression models, assuming Cohen’s f² = 0.02 and 80% power, 872 participants were required. For negative binomial regression assuming a rate ratio of 1.3, 305 participants were required. All three side effect analyses were adequately powered. For the Cox model using Schoenfeld’s method and the observed hazard ratio of 0.73, approximately 320 infection events were required to achieve 80% power. With 82 observed events, the Cox analysis achieved just 29% power.

Standard diagnostic checks were performed for all models. Multicollinearity was assessed using Variance Inflation Factors, model-specific assumptions were tested (linearity of logit, proportional odds, proportional hazards), and overdispersion was evaluated for count models. Alternate model specifications were evaluated, including models with interaction terms and sensitivity analyses using different cycle phase definitions. Model selection was based on AIC/BIC comparison, with lower values indicating better fit. Key diagnostic results are reported alongside model output.

## Supplementary information

Supplementary Materials

## Data Availability

Aggregated data are available in Supplementary Table 12. The dataset upon which the analysis was performed is available on the Open Science Framework at: https://osf.io/8a237/overview?view_only=543183f446c347aa98974bd827123b8a. The full underlying database that supports the findings of this study was made available by Clue by BioWink GmbH. While it is deidentified, it cannot be made directly available to the reader. Researchers interested in gaining access to the underlying data can contact Clue by BioWink GmbH and establish a data use agreement with them.

## Code availability

Code used in the analysis is available on the Open Science Framework at: https://osf.io/8a237/overview?view_only=543183f446c347aa98974bd827123b8a.
