# Supplementary material for: Menstrual cycle phase and its association with COVID-19 vaccine outcomes among period tracking app users
Source: NPJ Womens Health. 2026 Jun 6;4(1):36. doi: 10.1038/s44294-026-00150-x (PMC13696426; doi:10.1038/s44294-026-00150-x)
Supplement: Supplementary file 1 [file 44294_2026_150_MOESM1_ESM.pdf]

Supplementary Table 1: Association of cycle phase at vaccination and any self-reported side effects - Sensitivity/Subgroup Analysis

Sensitivity/Sub-group Analysis	OR (98.3% CI)	p-value	p-value (adj)	Significant?
**18-24**	**2.18 (1.24-3.84)**	**0.001**	**0.003**	**Yes (*)**
25-34	1.13 (0.73-1.76)	0.492	1.000	No
35-44	1.17 (0.70-1.98)	0.460	1.000	No
**Not Obese**	**1.48 (1.05-2.08)**	**0.006**	**0.018**	**Yes (*)**
**Post-secondary education**	**1.37 (1.01-1.84)**	**0.012**	**0.037**	**Yes (*)**
**No medical conditions**	**1.41 (1.00-1.98)**	**0.015**	**0.046**	**Yes (*)**
**Never smokers**	**1.54 (1.09-2.16)**	**0.003**	**0.008**	**Yes (*)**
White only	*1.32 (0.94-1.84)*	*0.048*	*0.143*	*Nominal (†)*
mRNA only	1.25 (0.93-1.69)	0.073	0.220	No
Pfizer only	1.34 (0.93-1.92)	0.054	0.162	No
Complete Cases	*1.34 (1.00-1.79)*	*0.017*	*0.051*	*Nominal (†)*
**No Copper Coil**	**1.40 (1.04-1.89)**	**0.007**	**0.021**	**Yes (*)**
Not menstruating	*1.34 (0.98-1.83)*	*0.025*	*0.074*	*Nominal (†)*
Not periovulatory	*1.29 (0.95-1.75)*	*0.049*	*0.146*	*Nominal (†)*
**Not perimenstrual**	**1.45 (1.05-2.00)**	**0.006**	**0.017**	**Yes (*)**
Systemic side effects	*1.33 (0.99-1.77)*	*0.021*	*0.062*	*Nominal (†)*

Supplementary Table 2: Self-reported side effect and infection outcomes for 18-24 age group and full sample by menstrual cycle phase at first COVID-19 vaccination

	**Luteal phase comparison**			**Follicular phase comparison**		
**Characteristic**	**Full sample** N = 714[1]	**Young (18-24)** N = 169[1]	**p-value**[2]	**Full sample** N = 760[1]	**Young (18-24)** N = 175[1]	**p-value**[2]
**Presence of side effects**	502 (70%)	97 (57%)	0.001	577 (76%)	131 (75%)	0.8
**Severity of side effects**			0.012			0.9
None	212 (30%)	72 (43%)		183 (24%)	44 (25%)	
Weak	324 (45%)	59 (35%)		386 (51%)	92 (53%)	
Moderate	123 (17%)	27 (16%)		111 (15%)	23 (13%)	
Severe	55 (7.7%)	11 (6.5%)		80 (11%)	16 (9.1%)	
**COVID-19 infection**	46 (6.4%)	12 (7.1%)	0.8	36 (4.7%)	11 (6.3%)	0.4
**Time to COVID-19** (days)	164 (107, 207)	197 (153, 227)	0.2	200 (140, 237)	186 (71, 235)	0.5

[1]P-values compare Young Age Group (18-24) vs Full Sample within each cycle phase

[2]Pearson's Chi-squared test; Wilcoxon rank sum test

Supplementary Table 3: Participant characteristics by age group

Characteristic	Overall<br>N = 1,474[1]	18-24<br>N = 344[1]	25-34<br>N = 639[1]	35-44<br>N = 491[1]	p-value[2]
**BMI category**					<0.001
Healthy weight	706 (48%)	202 (59%)	319 (50%)	185 (38%)	
Overweight	339 (23%)	73 (21%)	150 (23%)	116 (24%)	
Obese	429 (29%)	69 (20%)	170 (27%)	190 (39%)	
**Residence**					0.048
USA	1,046 (71%)	231 (67%)	448 (70%)	367 (75%)	
Other	428 (29%)	113 (33%)	191 (30%)	124 (25%)	
**Ethnicity**					<0.001
White	1,087 (74%)	218 (64%)	480 (76%)	389 (80%)	
Other	379 (26%)	125 (36%)	155 (24%)	99 (20%)	
**Income category**					<0.001
Lower income	341 (23%)	130 (38%)	154 (24%)	57 (12%)	
Middle income	573 (39%)	93 (27%)	277 (43%)	203 (41%)	
Higher income	465 (32%)	73 (21%)	184 (29%)	208 (42%)	
Don't know	95 (6.4%)	48 (14%)	24 (3.8%)	23 (4.7%)	
**Education**					<0.001
High school or less	105 (7.1%)	57 (17%)	33 (5.2%)	15 (3.1%)	
More than high school	1,369 (93%)	287 (83%)	606 (95%)	476 (97%)	
**Parity**					<0.001
Nulli-parity	1,236 (94%)	344 (100%)	602 (96%)	290 (83%)	
Parity	83 (6.3%)	0 (0%)	22 (3.5%)	61 (17%)	
Multi-parity	0 (0%)	0 (0%)	0 (0%)	0 (0%)	
Grand-parity	0 (0%)	0 (0%)	0 (0%)	0 (0%)	
**Medical condition(s): Yes (vs No)**					<0.001
Has medical condition(s)	435 (30%)	38 (11%)	181 (28%)	216 (44%)	
No medical condition	1,039 (70%)	306 (89%)	458 (72%)	275 (56%)	
**Smoking status**					0.6
Never	1,034 (70%)	239 (69%)	437 (68%)	358 (73%)	
Rarely	273 (19%)	66 (19%)	125 (20%)	82 (17%)	
Often	167 (11%)	39 (11%)	77 (12%)	51 (10%)	
**Vax1 type**					0.2
mRNA	1,283 (87%)	302 (89%)	563 (88%)	418 (85%)	
Other	185 (13%)	37 (11%)	76 (12%)	72 (15%)	
**Presence of side effects**	1,079 (73%)	228 (66%)	474 (74%)	377 (77%)	0.003
**Side effect severity**					0.014
None	395 (27%)	116 (34%)	165 (26%)	114 (23%)	
Weak	710 (48%)	151 (44%)	319 (50%)	240 (49%)	
Moderate	234 (16%)	50 (15%)	91 (14%)	93 (19%)	
Severe	135 (9.2%)	27 (7.8%)	64 (10%)	44 (9.0%)	
**Number of side effects**	2 (0, 4)	2 (0, 3)	2 (0, 4)	2 (1, 3)	0.11
**Covid-19 infection**	82 (5.6%)	23 (6.7%)	41 (6.4%)	18 (3.7%)	0.079
**Time to covid (days)**	184 (123, 233)	195 (125, 235)	167 (116, 203)	193 (153, 238)	0.4
**Survey duration (seconds)**	497 (392, 720)	508 (384, 797)	508 (393, 728)	480 (394, 691)	0.5
**cycle length (days)**	28 (27, 31)	30 (28, 32)	29 (27, 31)	28 (26, 29)	<0.001
**period length (days)**					0.007
1	68 (4.6%)	10 (2.9%)	32 (5.0%)	26 (5.3%)	
2	97 (6.6%)	13 (3.8%)	44 (6.9%)	40 (8.1%)	
3	261 (18%)	53 (15%)	106 (17%)	102 (21%)	
4	458 (31%)	105 (31%)	194 (30%)	159 (32%)	
5	349 (24%)	95 (28%)	145 (23%)	109 (22%)	
6	168 (11%)	51 (15%)	79 (12%)	38 (7.7%)	
7	64 (4.3%)	15 (4.4%)	34 (5.3%)	15 (3.1%)	
8	9 (0.6%)	2 (0.6%)	5 (0.8%)	2 (0.4%)	

[1]n (%); Median (Q1, Q3)
[2]Pearson's Chi-squared test; Kruskal-Wallis rank sum test

Supplementary Table 4: Self-reported side effect presence, severity, and number following first COVID-19 vaccination. Binary logistic, Ordinal logistic and Negative binomial multivariable regression model results

	A. Presence			B. Severity			C. Number		
**Characteristic**[1]	**OR**[1]	**95% CI**[1]	**p-value**[1]	**OR**[1]	**95% CI**[1]	**p-value**[1]	**IRR**[1]	**95% CI**[1]	**p-value**[1]
**Age group (per 10 years)**			**0.002**			0.070			0.3
25-34	—	—		—	—		—	—	
18-24	0.65	0.48, 0.89		0.80	0.61, 1.03		0.91	0.80, 1.03	
35-44	1.29	0.94, 1.78		1.15	0.89, 1.47		1.08	0.87, 1.10	
**Cycle length**	1.05	1.00, 1.09	0.030						
**Vax1 type**			**<0.001**			<0.001			<0.001
mRNA	—	—		—	—		—	—	
Other	2.32	1.54, 3.65		5.22	3.88, 7.02		1.71	1.50, 1.94	
**BMI category**			0.9			0.7			0.7
Healthy weight	—	—		—	—		—	—	
Overweight	1.05	0.78, 1.42		1.09	0.85, 1.39		1.05	0.93, 1.18	
Obese	0.96	0.72, 1.27		0.96	0.76, 1.22		1.02	0.91, 1.14	
**Medical condition(s):**			0.3			>0.9			0.8
Has medical condition(s)	—	—		—	—		—	—	
No medical condition	1.16	0.88, 1.51		0.99	0.80, 1.24		0.99	0.89, 1.10	
**Parity** (yes/no)	0.79	0.54, 1.14	0.2	0.80	0.59, 1.08	0.14	0.93	0.80, 1.08	0.3
**Smoking status**			0.060			0.11			0.041
Never	—	—		—	—		—	—	
Rarely	0.78	0.58, 1.06		0.83	0.64, 1.07		0.92	0.81, 1.04	
Often	1.34	0.91, 2.03		1.23	0.90, 1.67		1.16	1.00, 1.34	
**Income category**			0.7			0.2			0.6
Lower income	—	—		—	—		—	—	
Middle income	1.01	0.73, 1.38		1.16	0.89, 1.51		1.00	0.88, 1.14	
Higher income	1.19	0.84, 1.67		1.36	1.03, 1.79		1.08	0.94, 1.23	
Don't know	1.08	0.65, 1.84		1.18	0.76, 1.82		0.99	0.80, 1.22	
**Education**			0.2			0.3			0.8
High school or less	—	—		—	—		—	—	
More than high school	1.31	0.83, 2.05		1.22	0.83, 1.81		0.97	0.80, 1.18	
**Ethnicity**			0.8			0.6			0.7
White	—	—		—	—		—	—	
Other	0.97	0.74, 1.27		1.06	0.85, 1.33		1.02	0.92, 1.14	

[1]Note: Bold values indicate p < 0.0167 (Bonferroni correction for 3 models). CIs calculated at the 98.33% level.
(A) Binary logistic regression; OR = Odds Ratio. N = 1474; AIC = 1686.8
(B) Ordinal regression; OR = Odds Ratio. N = 1474; AIC = 3449.6
(C) Negative binomial regression; IRR = Incidence Rate Ratio. N = 1474; AIC = 5668.2
Abbreviations: CI = Confidence Interval, IRR = Incidence Rate Ratio, OR = Odds Ratio

Supplementary Table 5: Association of cycle phase at vaccination and self-reported side effect severity - Sensitivity/Subgroup Analysis

**Sensitivity Analysis/Sub-group**	**OR (98.3% CI)**	**p-value**	**p-value (adj)**	**Significant?**
18-24	*1.57 (0.97-2.57)*	***0.026***	*0.078*	*Nominal (†)*
25-34	1.22 (0.85-1.75)	0.181	0.544	No
35-44	0.98 (0.65-1.46)	0.886	1.000	No
Not Obese	*0.79 (0.59-1.04)*	***0.037***	*0.112*	*Nominal (†)*
Post-secondary education	0.82 (0.64-1.05)	0.052	0.155	No
No medical conditions	0.80 (0.61-1.06)	0.056	0.169	No
Never smokers	*0.78 (0.59-1.03)*	***0.030***	*0.089*	*Nominal (†)*
White only	0.84 (0.64-1.10)	0.129	0.386	No
mRNA only	0.87 (0.68-1.13)	0.201	0.603	No
Pfizer only	0.80 (0.58-1.09)	0.086	0.258	No
Complete Cases	0.52 (0.20-1.39)	0.113	0.339	No
No Copper Coil	*0.81 (0.63-1.03)*	***0.037***	*0.112*	*Nominal (†)*
Not menstruating	0.83 (0.64-1.06)	0.071	0.214	No
Not periovulatory	0.85 (0.66-1.10)	0.129	0.386	No
Not perimenstrual	*0.77 (0.59-1.01)*	***0.022***	*0.065*	*Nominal (†)*
Systemic side effects	*0.82 (0.64-1.04)*	***0.049***	*0.146*	*Nominal (†)*

Supplementary Table 6: Association of cycle phase at vaccination and self-reported side effect number - Sensitivity/Subgroup Analysis

**Sensitivity Analysis**	**IRR (98.3% CI)**	**p-value**	**p-value (adj)**	**Significant?**
18-24	1.24 (0.94-1.65)	0.067	0.202	No
25-34	1.12 (0.94-1.34)	0.121	0.363	No
35-44	0.93 (0.77-1.13)	0.389	1.000	No
Not Obese	0.90 (0.78-1.03)	0.067	0.201	No
Post-secondary education	0.91 (0.81-1.03)	0.072	0.216	No
No medical conditions	0.91 (0.79-1.05)	0.110	0.330	No
Never smokers	0.90 (0.78-1.03)	0.070	0.211	No
White only	0.93 (0.81-1.06)	0.201	0.604	No
mRNA only	0.96 (0.84-1.09)	0.418	1.000	No
Pfizer only	0.90 (0.77-1.06)	0.134	0.402	No
Complete Cases	0.79 (0.48-1.30)	0.251	0.753	No
No Copper Coil	0.91 (0.81-1.03)	0.081	0.242	No
Not menstruating	0.93 (0.81-1.05)	0.156	0.468	No
Not periovulatory	0.93 (0.82-1.06)	0.190	0.570	No
Not perimenstrual	0.91 (0.79-1.04)	0.093	0.279	No
Systemic side effects	0.91 (0.80-1.03)	0.067	0.201	No

Supplementary Table 7: COVID-19 infection risk by menstrual cycle phase at first vaccination: Cox proportional hazards regression results

**Characteristic**[1]	**HR**[1]	**95% CI**[1]	**p-value**[1]
Cycle phase (14-day luteal)			
Luteal	—	—	
Follicular	0.73	0.47, 1.13	0.2
Second vaccine status	0.39	0.22, 0.70	**0.001**
Age group (per 10 years)			
25-34	—	—	
18-24	1.22	0.72, 2.04	0.5
35-44	0.53	0.30, 0.93	**0.026**
Medical condition(s):			
No medical condition	—	—	
Has medical condition(s)	1.13	0.69, 1.86	0.6

[1]HR = Hazard Ration. Bold valued indicate p <0.05. Cox proportional hazards model. N = 1474Events N =82; AIC = 1121.5; Wald test = 20.228; Wald test P-value = 0.001; Log-Likelihood = -555.772; LR test =19.063; C-index =0.641; R-squared =0.331

Abbreviations: CI = Confidence Interval, HR = Hazard Ratio

Supplementary Table 8: Alternative cycle phase estimation model results

Cycle Phase	Analysis	Effect	Effect (98.3% CI)	P (raw)	P (adj)	Significant?	AIC
**Standard (14-day luteal)**	**Presence**	**OR**	**1.35 (1.01-1.79)**	**0.013**	**0.038**	**Yes (*)**	**1707.7**
Standard (14-day luteal)	Severity	OR	1.21 (0.95-1.52)	0.056	0.167	No	3587.1
Standard (14-day luteal)	Count	RR	1.08 (0.96-1.22)	0.109	0.326	No	5779.1
Standard (12-day luteal)	Presence	OR	1.22 (0.92-1.62)	0.099	0.298	No	1711.3
Standard (12-day luteal)	Severity	OR	1.15 (0.91-1.46)	0.156	0.467	No	3588.8
Standard (12-day luteal)	Count	RR	1.05 (0.93-1.18)	0.345	1.000	No	5780.8
Standard (13-day luteal)	*Presence*	*OR*	*1.29 (0.97-1.71)*	***0.032***	*0.096*	*Nominal (†)*	*1709.4*
Standard (13-day luteal)	Severity	OR	1.21 (0.96-1.53)	0.053	0.158	No	3587.0
Standard (13-day luteal)	Count	RR	1.07 (0.95-1.21)	0.151	0.453	No	5779.6
Age-adjusted	Presence	OR	1.21 (0.91-1.61)	0.113	0.339	No	1711.5
Age-adjusted	Severity	OR	1.15 (0.91-1.45)	0.168	0.504	No	3588.9
Age-adjusted	Count	RR	1.05 (0.94-1.19)	0.299	0.896	No	5780.6
Cycle-length-adjusted	Presence	OR	1.22 (0.92-1.63)	0.089	0.268	No	1711.1
Cycle-length-adjusted	Severity	OR	1.16 (0.92-1.47)	0.133	0.398	No	3588.5
Cycle-length-adjusted	Count	RR	1.05 (0.93-1.18)	0.308	0.923	No	5780.6

Note: OR = Odds Ratio, RR = Rate Ratio, HR = Hazard Ratio. Best fitting models (lowest AIC) for each analysis type highlighted in green. Significant results ($p < 0.05$) in bold.

Supplementary Table 9: Table showing predicted probability of self-reported side effects throughout menstrual cycle, using Phase Aligned Cycle Timing

**Phase Aligned Cycle Timing (0.05 increments)**	**Cycle Phase**	**Predicted Probability**	**Standard Error**
0.000	*Menses Onset*	0.833	0.046
0.050	*Follicular Phase*	0.699	0.054
0.100	*Follicular Phase*	0.783	0.050
0.150	*Follicular Phase*	0.831	0.049
0.200	*Follicular Phase*	0.767	0.046
0.250	*Follicular Phase*	0.767	0.050
0.300	*Follicular Phase*	0.697	0.057
0.350	*Follicular Phase*	0.667	0.060
0.400	*Follicular Phase*	0.761	0.051
0.450	*Follicular Phase*	0.750	0.050
0.500	*Imputed Ovulation*	0.810	0.052
0.550	*Luteal Phase*	0.649	0.049
0.600	*Luteal Phase*	0.625	0.071
0.650	*Luteal Phase*	0.643	0.049
0.700	*Luteal Phase*	0.700	0.055
0.750	*Luteal Phase*	0.692	0.049
0.800	*Luteal Phase*	0.739	0.065
0.850	*Luteal Phase*	0.750	0.043
0.900	*Luteal Phase*	0.860	0.050
0.950	*Luteal Phase*	0.717	0.043

Supplementary Figure 1: Probability of self-reported side effects across the menstrual cycle in the 18-24 age group

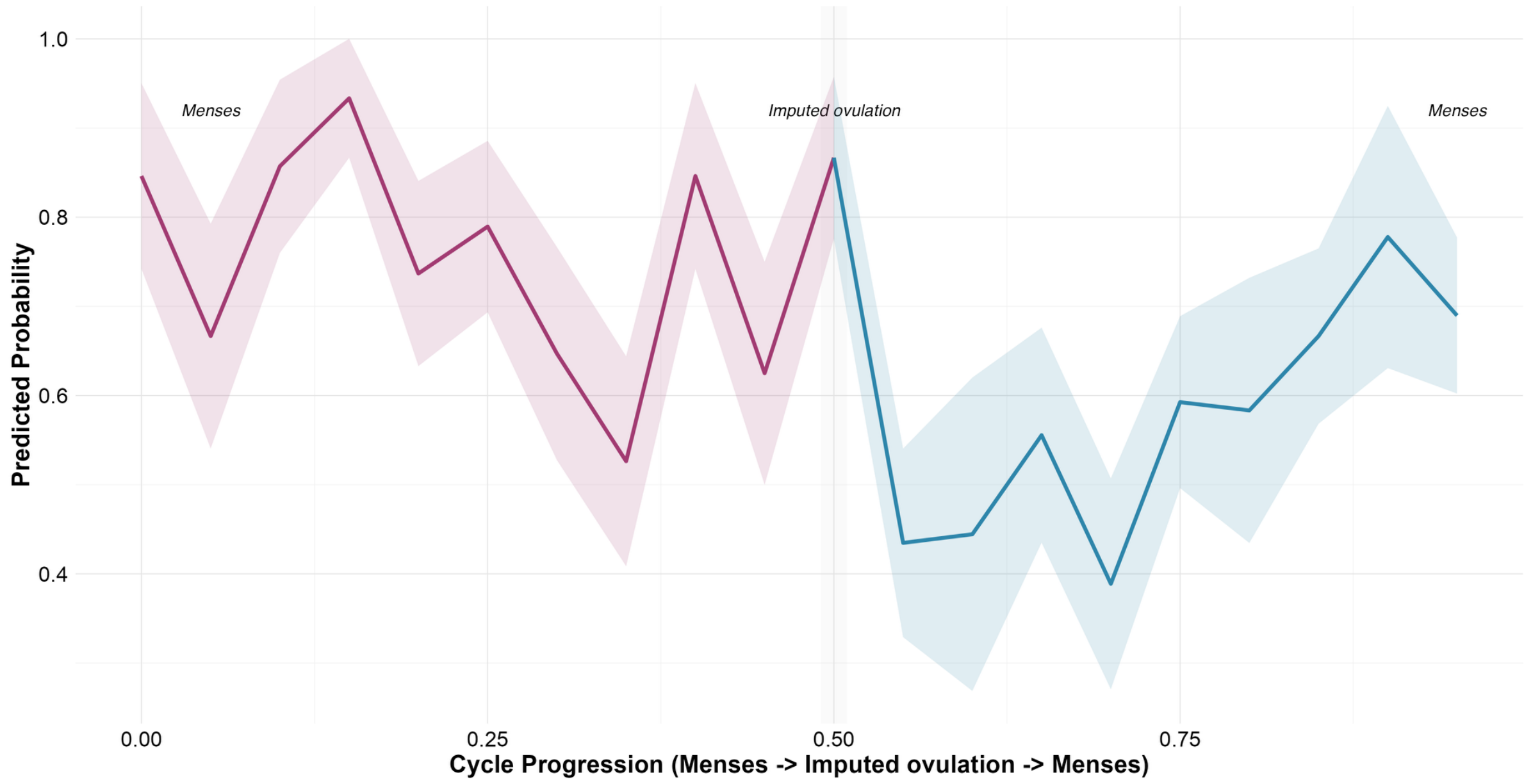

Note: Plot shows probability of self-reporting side effects following first COVID-19 vaccination across a standardised menstrual cycle using Phase Aligned Cycle Timing in 18-24 age group. Menses onset is anchored at 0 and 1, with ovulation at 0.50. Points are plotted at 0.05 increments to show how the probability of self-reporting side effects fluctuates across the cycle. Shaded areas ±1 SE.

Supplementary Table 10: Table showing predicted probability of self-reported side effects throughout menstrual cycle, using Phase Aligned Cycle Timing in 18-24 age-group

**Phase Aligned Cycle Timing (0.05 increments)**	**Cycle Phase**	**Probability (Mean)**	**Standard Error**
0.000	*Menses Onset*	0.846	0.104
0.050	*Follicular Phase*	0.667	0.126
0.100	*Follicular Phase*	0.857	0.097
0.150	*Follicular Phase*	0.933	0.067
0.200	*Follicular Phase*	0.737	0.104
0.250	*Follicular Phase*	0.789	0.096
0.300	*Follicular Phase*	0.647	0.119
0.350	*Follicular Phase*	0.526	0.118
0.400	*Follicular Phase*	0.846	0.104
0.450	*Follicular Phase*	0.625	0.125
0.500	*Imputed Ovulation*	0.867	0.091
0.550	*Luteal Phase*	0.435	0.106
0.600	*Luteal Phase*	0.444	0.176
0.650	*Luteal Phase*	0.556	0.121
0.700	*Luteal Phase*	0.389	0.118
0.750	*Luteal Phase*	0.593	0.096
0.800	*Luteal Phase*	0.583	0.149
0.850	*Luteal Phase*	0.667	0.098
0.900	*Luteal Phase*	0.778	0.147
0.950	*Luteal Phase*	0.690	0.087

Supplementary Figure 2: Directed Acyclic Graph – Vaccination cycle-phase and self-reported side effects

Shortest adjustment set: Age, BMI, comorbidities, ethnicity, smoking

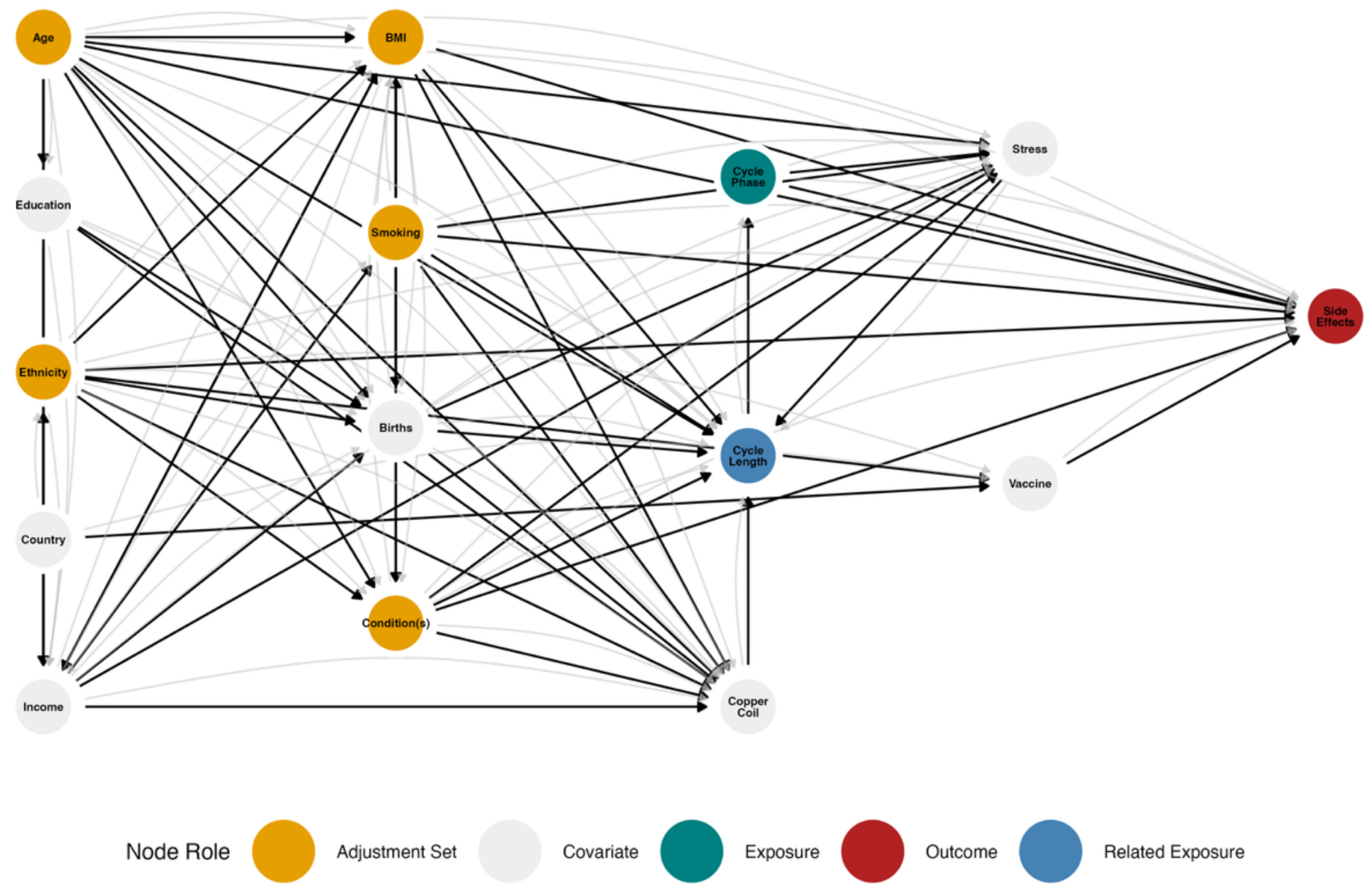

Supplementary Figure 3: Directed Acyclic Graph – Vaccination cycle-phase and COVID-19 infection

Shortest adjustment set: Age, BMI, comorbidities, ethnicity, smoking

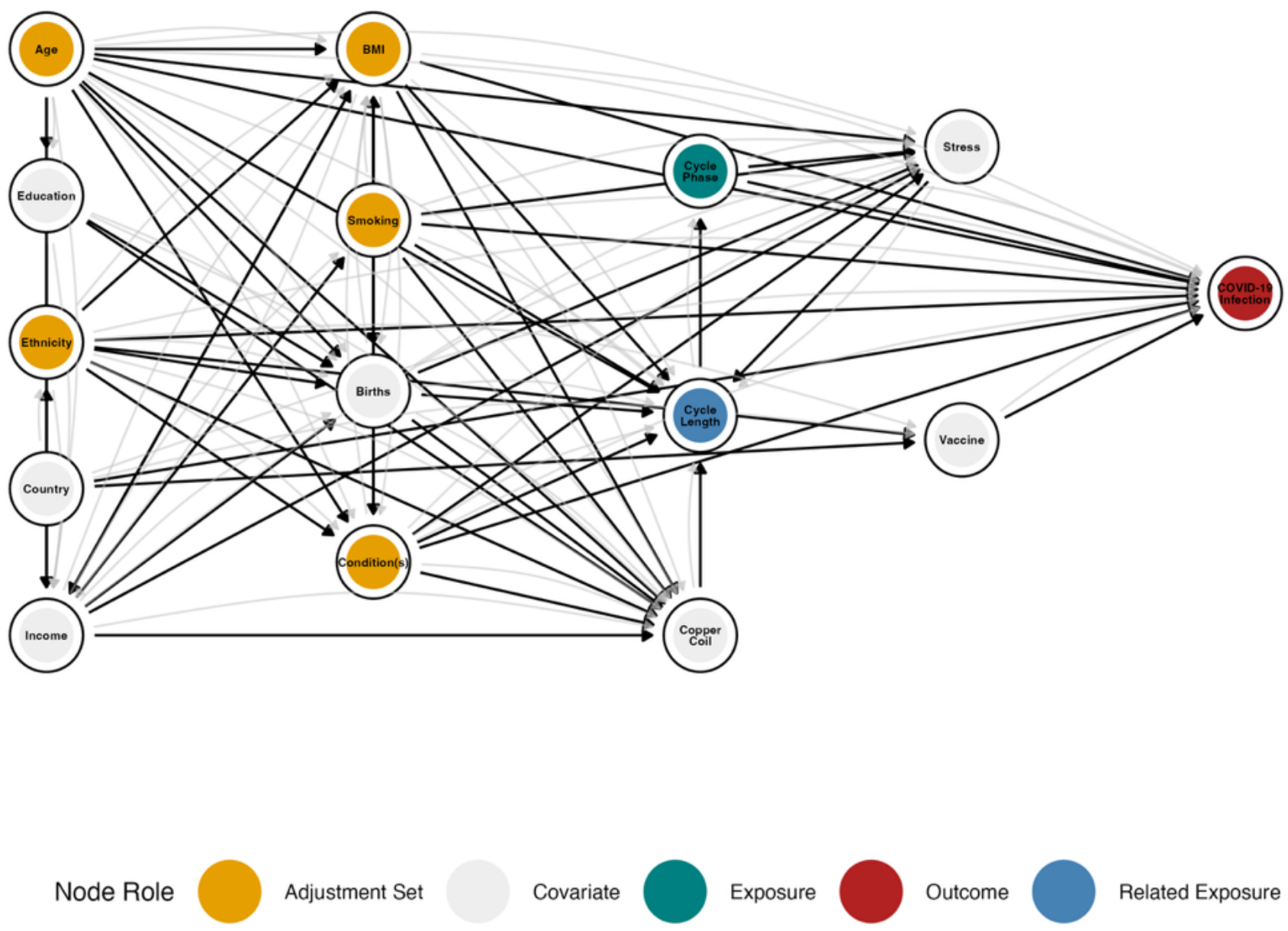

Supplementary Table 11: Self-reported menstrual cycle changes by menstrual cycle phase at first COVID-19 vaccination

**Characteristic**	**Overall**<br>N = 1,474[1]	**Follicular**<br>N = 760[1]	**Luteal**<br>N = 714[1]	**p-value**[2]
**Heavier flow, n (%)**	100 (6.8%)	51 (6.7%)	49 (6.9%)	0.99
**Longer bleed, n (%)**	71 (4.8%)	31 (4.1%)	40 (5.6%)	0.21
**Longer cycle, n (%)**	58 (3.9%)	30 (3.9%)	28 (3.9%)	1.00
**Cramps, n (%)**	55 (3.7%)	26 (3.4%)	29 (4.1%)	0.61
**Irregular cycle, n (%)**	49 (3.3%)	19 (2.5%)	30 (4.2%)	0.09
**Shorter cycle, n (%)**	42 (2.8%)	18 (2.4%)	24 (3.4%)	0.32
**Shorter bleed, n (%)**	22 (1.5%)	10 (1.3%)	12 (1.7%)	0.72
**Lighter flow, n (%)**	16 (1.1%)	8 (1.1%)	8 (1.1%)	1.00
**Spotting, n (%)**	16 (1.1%)	5 (0.7%)	11 (1.5%)	0.17
**Other, n (%)**	15 (1.0%)	9 (1.2%)	6 (0.8%)	0.69
**Skipped/stopped, n (%)**	8 (0.5%)	3 (0.4%)	5 (0.7%)	0.66

[1]n (%); Median (Q1, Q3) for continuous variables

[2]Chi-square test for categorical variables; Wilcoxon rank-sum test for continuous variables

Supplementary Figure 4: Period and Pandemic Survey

Periods and the pandemic

---

1. **Please confirm that you have read the information above and agree with the following statements:** *

- ☐ I have read the information and voluntarily agree to participate in this study
- ☐ I am 18 years of age or older
- ☐ I experience menstrual periods (or withdrawal bleeds)
- ☐ I have not been pregnant or breastfeeding since January 2020

2. **How old are you?**

18
19
20
21
22
23
24
25
26
27
28
29
30
31
32
33
34
35
36
37
38
39
40
41
42
43
44
45
46
47
48
49
50
51
52
53
54
55
56
57
58
59
60
61
62
63
64
65
66
67
68
69
70

Clue_ID **Action: Hidden Value**
Value: [url("id")]

(untitled)

Page exit logic: Skip / Disqualify Logic
IF: #3 Question "

Where do you currently live?

" is one of the following answers ("Other") **THEN:** Disqualify and display:
Thank you for supporting Clue research. For this survey, we're focusing on just a few countries, but we will have more surveys for you in the future!

LOGIC Show/hide trigger exists.

3. **Where do you currently live?**

- United States of America
- United Kingdom
- Australia
- Canada
- Other

(untitled)

Page exit logic: Disqualify respondents from other countries
IF: #3 Question "

Where do you currently live?

" is not one of the following answers ("United States of America","United Kingdom","Australia","Canada")**THEN:** Disqualify and display:
This specific study focuses on people living in Australia, Canada, the United States of America and the United Kingdom. Thank you for your interest in contributing to our research!

LOGIC Hidden unless: #3 Question "

Where do you currently live?

" is one of the following answers ("United Kingdom")

4. **Which country do you live in?**

- England
- Scotland
- Wales
- Northern Ireland
- Other - Write In

LOGIC Hidden unless: #3 Question "

Where do you currently live?

" is one of the following answers ("United States of America")

5. **Which state do you live in?**

- Alabama
- Alaska
- Arizona
- Arkansas
- California
- Colorado
- Connecticut
- Delaware
- Florida
- Georgia
- Hawaii
- Idaho
- Illinois
- Indiana
- Iowa
- Kansas
- Kentucky
- Louisiana
- Maine
- Maryland
- Massachusetts
- Michigan
- Minnesota
- Mississippi
- Missouri
- Montana
- Nebraska
- Nevada
- New Hampshire
- New Jersey
- New Mexico
- New York
- North Carolina
- North Dakota
- Ohio
- Oklahoma
- Oregon
- Pennsylvania
- Puerto Rico
- Rhode Island
- South Carolina
- South Dakota
- Tennessee
- Texas
- Utah
- Vermont
- Virginia
- Washington
- Washington, D.C.
- West Virginia
- Wisconsin
- Wyoming
- Other US territory

LOGIC Hidden unless: #3 Question "
Where do you currently live?

" is one of the following answers ("Canada")

6. **Which province do you live in?**

- Alberta
- British Columbia
- Manitoba
- New Brunswick
- Newfoundland and Labrador
- Nova Scotia
- Ontario
- Prince Edward Island
- Quebec
- Saskatchewan
- Other - Write In

LOGIC Hidden unless: #3 Question "
Where do you currently live?

" is one of the following answers ("Australia")

7. **Which state or territory do you live in?**

- Northern Territory
- New South Wales
- Australian Capital Territory
- Queensland
- South Australia
- Tasmania
- Victoria
- Western Australia
- External Territories
- Other - Write In

LOGIC Show/hide trigger exists.

8. **We need to ask for your weight - do you prefer kilograms (kg), pounds (lbs) or stones?**

- kg
- lbs
- stones

LOGIC Hidden unless: #8 Question "**We need to ask for your weight - do you prefer kilograms (kg), pounds (lbs) or stones?** is one of the following answers ("kg")

9. **How much do you weigh in kg?**

30
31
32
33
34
35
36
37
38
39
40
41
42
43
44
45
46
47
48
49
50
51
52
53
54
55
56
57
58
59
60
61
62
63
64
65
66
67
68
69
70
71
72
73
74
75
76
77
78
79
80
81
82
83

84
85
86
87
88
89
90
91
92
93
94
95
96
97
98
99
100
101
102
103
104
105
106
107
108
109
110
111
112
113
114
115
116
117
118
119
120+
I don't know
Prefer not to say

LOGIC Hidden unless: #8 Question "**We need to ask for your weight - do you prefer kilograms (kg), pounds (lbs) or stones?** is one of the following answers ("lbs")

10. **How much do you weigh in pounds (lbs)?**

66
67
68
69
70
71
72
73
74
75
76
77
78
79
80
81
82
83
84
85
86
87
88
89
90
91

92
93
94
95
96
97
98
99
100
101
102
103
104
105
106
107
108
109
110
111
112
113
114
115
116
117
118
119
120
121
122
123
124
125
126
127
128
129
130
131
132
133
134
135
136
137
138
139
140
141
142
143
144
145
146
147
148
149
150
151
152
153
154
155
156
157
158
159
160
161
162
163
164
165

239
240
241
242
243
244
245
246
247
248
249
250
251
252
253
254
255
256
257
258
259
260
261
262
263
264
265
265+
I don't know
Prefer not to say

LOGIC Hidden unless: #8 Question "**We need to ask for your weight - do you prefer kilograms (kg), pounds (lbs) or stones?** is one of the following answers ("stones")

11. **How much do you weigh in stones?**

6s
6s1
6s2
6s3
6s4
6s5
6s6
6s7
6s8
6s9
6s10
6s11
6s12
6s13
7s
7s1
7s2
7s3
7s4
7s5
7s6
7s7
7s8
7s9
7s10
7s11
7s12
7s13
8s
8s1
8s2
8s3
8s4
8s5
8s6

8s7
8s8
8s9
8s10
8s11
8s12
8s13
9s
9s1
9s2
9s3
9s4
9s5
9s6
9s7
9s8
9s9
9s10
9s11
9s12
9s13
10s
10s1
10s2
10s3
10s4
10s5
10s6
10s7
10s8
10s9
10s10
10s11
10s12
10s13
11s
11s1
11s2
11s3
11s4
11s5
11s6
11s7
11s8
11s9
11s10
11s11
11s12
11s13
12s
12s1
12s2
12s3
12s4
12s5
12s6
12s7
12s8
12s9
12s10
12s11
12s12
12s13
13s
13s1
13s2
13s3
13s4
13s5
13s6
13s7
13s8
13s9
13s10

13s10
13s11
13s12
13s13
14s
14s1
14s2
14s3
14s4
14s5
14s6
14s7
14s8
14s9
14s10
14s11
14s12
14s13
15s
15s1
15s2
15s3
15s4
15s5
15s6
15s7
15s8
15s9
15s10
15s11
15s12
15s13
16s
16s1
16s2
16s3
16s4
16s5
16s6
16s7
16s8
16s9
16s10
16s11
16s12
16s13
17s
17s1
17s2
17s3
17s4
17s5
17s6
17s7
17s8
17s9
17s10
17s11
17s12
17s13
18s
18s1
18s2
18s3
18s4
18s5
18s6
18s7
18s8
18s9
18s10
18s11
18s12
18s13

19s
19s1
19s2
19s3
19s4
19s5
19s6
19s7
19s8
19s9
19s10
19s11
19s12
19s13
20s
20s1
20s2
20s3
20s4
20s5
20s6
20s7
20s8
20s9
20s10
20s11
20s12
20s13
I don't know
Prefer not to say

LOGIC Show/hide trigger exists.

12. **We need to ask for your height - do you prefer centimetres (cm) or feet & inches?**

- cm
- feet & inches

LOGIC Hidden unless: #12 Question "**We need to ask for your height - do you prefer centimetres (cm) or feet & inches?**" is one of the following answers ("cm")

13. **What is your height in cm?**

130
131
132
133
134
135
136
137
138
139
140
141
142
143
144
145
146
147
148
149
150
151
152
153

154
155
156
157
158
159
160
161
162
163
164
165
166
167
168
169
170
171
172
173
174
175
176
177
178
179
180
181
182
183
184
185
186
187
188
189
190
191
192
193
194
195
196
197
198
199
200
201
202
203
204
205
206
207
208
209
210
>210

LOGIC Hidden unless: #12 Question "**We need to ask for your height - do you prefer centimetres (cm) or feet & inches?**" is one of the following answers ("feet & inches")

14. **What is your height in feet and inches?**

4' 0
4' 1"
4' 2
4' 3
4' 4
4' 5
4' 6
4' 7
4' 8
4' 9
4' 10
4' 11
5' 0
5' 1"
5' 2
5' 3
5' 4
5' 5
5' 6
5' 7
5' 8
5' 9
5' 10
5' 11
6' 0
6' 1"
6' 2
6' 3
6' 4
6' 5
6' 6
6' 7
6' 8
6' 9
6' 10
6' 11

15. **What is the highest level of education you have completed?**

- Less than high school
- Some high school
- High school diploma (A-levels, BTEC, AP)
- Some college or University
- Undergraduate degree (e.g. Bachelor's degree)
- Post-graduate degree (e.g. Master's, PhD, MD, JD)

16. **How many children have you given birth to?**

- 0
- 1
- 2
- 3
- 4
- 5+

LOGIC Show/hide trigger exists.

17. **Have you ever smoked tobacco or vaped e-cigarettes?** Select those that you have ever done

- Smoked tobacco
- Vaped e-cigarettes
- None

LOGIC Hidden unless: #17 Question "**Have you ever smoked tobacco or vaped e-cigarettes?**Select those that you have ever done" is one of the following answers ("Smoked tobacco")

18. **How often do you smoke tobacco?**

- I have not smoked in the past 2 years
- Several times a day
- Once a day
- Once a week
- Once a month
- Less than a few times per year

LOGIC Hidden unless: #17 Question "**Have you ever smoked tobacco or vaped e-cigarettes?**Select those that you have ever done" is one of the following answers ("Vaped e-cigarettes")

19. **How often do you vape e-cigarettes?**

- I have not vaped in the past 2 years
- Several times a day
- Once a day
- Once a week
- Once a month
- Less than a few times per year

LOGIC Show/hide trigger exists.

20. **Have you ever smoked cannabis?**

- No
- Yes

LOGIC Hidden unless: #20 Question "**Have you ever smoked cannabis?**" is one of the following answers ("Yes")

21. **How often do you smoke cannabis?**

- I do not currently smoke cannabis
- Several times a day
- Once a day
- Once a week
- Once a month
- Less than a few times a year

22. **What do you identify as?**

- Woman
- Man
- Genderqueer / Non-Binary
- Questioning/ Unsure
- Prefer not to answer
- Other (Please specify)

LOGIC Hidden unless: #3 Question "
Where do you currently live?
" is one of the following answers ("United States of America")

23. **How would you describe your ethnic identity?** (check all that apply)

- ☐ White or European American
- ☐ Hispanic, Latina or Latinx
- ☐ Black or African-American
- ☐ Asian or Asian-American
- ☐ Native American or Alaska native
- ☐ Native Hawaiian or other Pacific Islander
- ☐ Middle Eastern / North African
- ☐ Prefer not to say

LOGIC Hidden unless: #3 Question "
Where do you currently live?
" is one of the following answers ("United Kingdom")

24. **How would you describe your ethnic identity?** (check all that apply)

- ☐ White European
- ☐ Black British or Afro-Caribbean
- ☐ Asian or Asian British
- ☐ Middle Eastern or Middle Eastern British
- ☐ Roma/Gypsy/Traveller/Irish Traveller
- ☐ Prefer not to say

LOGIC Hidden unless: #3 Question "
Where do you currently live?

" is one of the following answers ("Canada")

25. **How would you describe your ethnic identity?** (check all that apply)

- ☐ First Nations, Indigenous, Aboriginal, or Native peoples
- ☐ Northern Asian (Chinese, Japanese, Korean)
- ☐ Southern Asian (India, Pakistan)
- ☐ Southeast Asian & Filipino
- ☐ Black Canadian, Caribbean Canadian, or African Canadian
- ☐ White (European ancestry)
- ☐ Arab, Middle Eastern, or North African
- ☐ Latin American
- ☐ Prefer not to say

LOGIC Hidden unless: #3 Question "
Where do you currently live?

" is one of the following answers ("Australia")

26. **How would you describe your ethnic identity?** (check all that apply)

- ☐ White
- ☐ Asian
- ☐ Black or African
- ☐ Aboriginal
- ☐ Middle Eastern
- ☐ Pacific Islander
- ☐ Prefer not to say

Hidden unless: #3 Question "
Where do you currently live?

" is one of the following answers ("United Kingdom")

27. **What is the total take-home pay** *(the money you have after all taxes and contributions have been deducted, also known as Net Income)* **earned by you and other people in your household (your partner/family)?** *If you live on campus or with your parents, and if you rely on your parents' income more than you rely on your own income, then indicate your parents' income.*

- Less than £13,680 (year) OR £1,140 (month)
- £13,680 to £22,140 (year) OR £1,140 to £1,840 (month)
- £22,140 to £29,250 (year) OR £1,840 to £2,440 (month)
- £29,250 to £39,400 (year) OR £2,440 to £3,280 (month)
- £39,400 to £76,140 (year) OR £3,280 to £6,340 (month)
- More than £76,140 (year) OR £6,340 (month)
- Don't know

Hidden unless: #3 Question "
Where do you currently live?

" is one of the following answers ("United States of America")

28. **What is the total take-home pay** *(the money you have after all taxes and contributions have been deducted, also known as Net Income)* **earned by you and other people in your household (your partner/family)?** *If you live on campus or with your parents, and if you rely on your parents' income more than you rely on your own income, then indicate your parents' income.*

- Less than $13,260 (year) OR $1,100 (month)
- $13,260 to $35,400 (year) OR $1,100 to $2,950 (month)
- $35,400 to $61,560 (year) OR $2,950 to $5,130 (month)
- $61,560 to $99,030 (year) OR $5,130 to $8,250 (month)
- $99,030 to $221,850 (year)OR $8,250 to $18,490 (month)
- More than $221,850 (year) OR $18,490 (month)
- Don't know

LOGIC Hidden unless: #3 Question "
Where do you currently live?

" is one of the following answers ("Canada")

29. **What is the total take-home pay** *(the money you have after all taxes and contributions have been deducted, also known as Net Income)* **earned by you and other people in your household (your partner/family)?** *If you live on campus or with your parents, and if you rely on your parents' income more than you rely on your own income, then indicate your parents' income.*

- Less than CA$25,430 (year) OR CA$2,119 (month)
- CA$25,430 to CA$52,010 (year) OR CA$2,119 to CA$4,330 (month)
- CA$52,010 to CA$71,150 (year) OR CA$4,330 to CA$5,930 (month)
- CA$71,150 to CA$95,200 (year) OR CA$5,930 to CA$7,930 (month)
- CA$95,200 to CA$162,960 (year) OR CA$7,930 to CA$13,580 (month)
- More than CA$162,960 (year) OR CA$13,580 (month)
- Don't know

LOGIC Hidden unless: #3 Question "
Where do you currently live?

" is one of the following answers ("Australia")

30. **What is the total take-home pay** *(the money you have after all taxes and contributions have been deducted, also known as Net Income)* **earned by you and other people in your household (your partner/family)?** *If you live on campus or with your parents, and if you rely on your parents' income more than you rely on your own income, then indicate your parents' income.*

- Less than $13,960 (year) OR $1,160 (month)
- $13,960 to $40,400 (year) OR $1,160 to $3,370 (month)
- $40,400 to $79,780 (year) OR $3,370 to $6,650 (month)
- $79,780 to $123,490 (year) OR $6,650 to $10,290(month)
- $123,490 to $218,840 (year) OR $10,290 to $18,240 (month)
- More than $218,840 (year) OR $18,240 (month)
- Don't know

31. **Have you been diagnosed with any of the following conditions?** (check all that apply)

- ☐ Endometriosis
- ☐ Polycystic ovary syndrome (PCOS)
- ☐ Thyroid disease
- ☐ Uterine polyps or fibroids
- ☐ Eating disorder (e.g. anorexia, bulimia, binge-eating...)
- ☐ Pelvic inflammatory disease
- ☐ Interstitial cystitis
- ☐ Diabetes
- ☐ Gynecological cancers
- ☐ Any other cancer
- ☐ Von Willebrand disease
- ☐ Abnormal pap smear
- ☐ Condition requiring immunosuppresive therapy
- ☐ None of the above
- ☐ Prefer not to answer

32. **How many people can you count on if you have significant personal problems?** This can include family, friends, coworkers, neighbors, etc.

- ○ None
- ○ 1–2
- ○ 3–5
- ○ 5+

33. **How easy is it to get practical help from neighbors** (e.g. someone who lives close to you but is not necessarily family/friends) **in case of urgent need?** For example, if you need to borrow money or a car, or if you need help coping with a crisis.

- ○ Very difficult
- ○ Difficult
- ○ Possible
- ○ Easy
- ○ Very easy

34. **How much interest and concern do people show in what you do?** *This includes your work, interests and/or hobbies.*

- None
- Little
- Uncertain
- Some
- A lot

LOGIC Show/hide trigger exists.

35. **Were you using a hormonal contraceptive or a copper IUD in January 2020?**

- Yes
- No

LOGIC Show/hide trigger exists. Hidden unless: #35 Question "**Were you using a hormonal contraceptive or a copper IUDin January 2020?** " is one of the following answers ("Yes")

36. **Select all the method(s) you were using in January 2020.**

- Combined oral contraceptive pill (e.g. Microgynon, Marvelon, Yasmin)
- Progestin only pill (e.g. Minipill)
- Contraceptive Injection (e.g. Depo-Provera, Sayana Press, Noristerat)
- Hormonal IUD/IUS/Coil (e.g. Mirena, Kyleena, Jaydess, Levosert or Skyla)
- Contraceptive patch (e.g. Ortho Evra, Xulane
- Vaginal ring (e.g. Nuvaring)
- Contraceptive Implant (e.g. Nexplanon)
- Copper IUD
- None of the above

LOGIC Show/hide trigger exists. Hidden unless: #36 Question "
Select all the method(s) you were using in January 2020.

" is one of the following answers ("Combined oral contraceptive pill (e.g. Microgynon, Marvelon, Yasmin)")

37. **Did you use the combined oral contraceptive pill for the entire pandemic (since 2020)?**

- Yes
- No

LOGIC Show/hide trigger exists. Hidden unless: #36 Question "
Select all the method(s) you were using in January 2020.

" is one of the following answers ("Progestin only pill (e.g. Minipill)")

38. **Did you use the progestin only pill for the entire pandemic (since 2020)?**

- Yes
- No

LOGIC Show/hide trigger exists. Hidden unless: #36 Question "
Select all the method(s) you were using in January 2020.

" is one of the following answers ("Contraceptive Injection (e.g. Depo-Provera, Sayana Press, Noristerat)")

39. **Did you use the contraceptive injection for the entire pandemic (since 2020)?**

- Yes
- No

LOGIC Show/hide trigger exists. Hidden unless: #36 Question "
Select all the method(s) you were using in January 2020.

" is one of the following answers ("Hormonal IUD/IUS/Coil (e.g. Mirena, Kyleena, Jaydess, Levosert or Skyla)")

40. **Did you use the hormonal IUD/IUS/Coil for the entire pandemic (since 2020)?**

- Yes
- No

LOGIC Show/hide trigger exists. Hidden unless: #36 Question "
Select all the method(s) you were using in January 2020.

" is one of the following answers ("Contraceptive patch (e.g. Ortho Evra, Xulane")

41. **Did you use the contraceptive patch for the entire pandemic (since 2020)?**

- Yes
- No

LOGIC Show/hide trigger exists. Hidden unless: #36 Question "
Select all the method(s) you were using in January 2020.

" is one of the following answers ("Vaginal ring (e.g. Nuvaring)")

42. **Did you use the vaginal ring for the entire pandemic (since 2020)?**

- Yes
- No

Show/hide trigger exists. Hidden unless: #36 Question "
Select all the method(s) you were using in January 2020.

" is one of the following answers ("Contraceptive Implant (e.g. Nexplanon)")

43. **Did you use the contraceptive implant for the entire pandemic (since 2020)?**

- Yes
- No

Show/hide trigger exists. Hidden unless: #36 Question "
Select all the method(s) you were using in January 2020.

" is one of the following answers ("Copper IUD")

44. **Did you use the copper IUD for the entire pandemic (since 2020)?**

- Yes
- No

Hidden unless: #37 Question "
Did you use the combined oral contraceptive pill for the entire pandemic (since 2020)?

" is one of the following answers ("No")

45. **When did you stop using the combined oral contraceptive pill?** *If you used it for more than one time period, when did you stop the first time?*

Jan 2020
Feb 2020
March 2020
April 2020
May 2020
June 2020
July 2020
August 2020
Sept 2020
Oct 2020
Nov 2020
Dec 2020
Jan 2021
Feb 2021
March 2021
April 2021
May 2021
June 2021
July 2021
August 2021
Sept 2021
Oct 2021
Nov 2021
Dec 2021

LOGIC Hidden unless: #38 Question "

Did you use the progestin only pill for the entire pandemic (since 2020)?

" is one of the following answers ("No")

46. **When did you stop using the progesterone only pill?** *If you used it for more than one time period, when did you stop the first time?*

Jan 2020
Feb 2020
March 2020
April 2020
May 2020
June 2020
July 2020
August 2020
Sept 2020
Oct 2020
Nov 2020
Dec 2020
Jan 2021
Feb 2021
March 2021
April 2021
May 2021
June 2021
July 2021
August 2021
Sept 2021
Oct 2021
Nov 2021
Dec 2021

LOGIC Hidden unless: #39 Question "

Did you use the contraceptive injection for the entire pandemic (since 2020)?

" is one of the following answers ("No")

47. **When did you stop using the contraceptive injection?** *If you used it for more than one time period, when did you stop the first time?*

Jan 2020
Feb 2020
March 2020
April 2020
May 2020
June 2020
July 2020
August 2020
Sept 2020
Oct 2020
Nov 2020
Dec 2020
Jan 2021
Feb 2021
March 2021
April 2021
May 2021
June 2021
July 2021
August 2021
Sept 2021
Oct 2021
Nov 2021
Dec 2021

LOGIC Hidden unless: #40 Question "
Did you use the hormonal IUD/IUS/Coil for the entire pandemic (since 2020)?

" is one of the following answers ("No")

48. **When did you stop using the hormonal IUD/IUS/Coil?** *If you used it for more than one time period, when did you stop the first time?*

Jan 2020
Feb 2020
March 2020
April 2020
May 2020
June 2020
July 2020
August 2020
Sept 2020
Oct 2020
Nov 2020
Dec 2020
Jan 2021
Feb 2021
March 2021
April 2021
May 2021
June 2021
July 2021
August 2021
Sept 2021
Oct 2021
Nov 2021
Dec 2021

LOGIC Hidden unless: #41 Question "
Did you use the contraceptive patch for the entire pandemic (since 2020)?

" is one of the following answers ("No")

49. **When did you stop using the contraceptive patch?** *If you used it for more than one time period, when did you stop the first time?*

Jan 2020
Feb 2020
March 2020
April 2020
May 2020
June 2020
July 2020
August 2020
Sept 2020
Oct 2020
Nov 2020
Dec 2020
Jan 2021
Feb 2021
March 2021
April 2021
May 2021
June 2021
July 2021
August 2021
Sept 2021
Oct 2021
Nov 2021
Dec 2021

LOGIC Hidden unless: #43 Question "
Did you use the contraceptive implant for the entire pandemic (since 2020)?

" is one of the following answers ("No")

50. **When did you stop using the contraceptive implant?** *If you used it for more than one time period, when did you stop the first time?*

Jan 2020
Feb 2020
March 2020
April 2020
May 2020
June 2020
July 2020
August 2020
Sept 2020
Oct 2020
Nov 2020
Dec 2020
Jan 2021
Feb 2021
March 2021
April 2021
May 2021
June 2021
July 2021
August 2021
Sept 2021
Oct 2021
Nov 2021
Dec 2021

LOGIC Hidden unless: #44 Question "
Did you use the copper IUD for the entire pandemic (since 2020)?

" is one of the following answers ("No")

51. **When did you stop using the copper IUD?** *If you used it for more than one time period, when did you stop the first time?*

Jan 2020
Feb 2020
March 2020
April 2020
May 2020
June 2020
July 2020
August 2020
Sept 2020
Oct 2020
Nov 2020
Dec 2020
Jan 2021
Feb 2021
March 2021
April 2021
May 2021
June 2021
July 2021
August 2021
Sept 2021
Oct 2021
Nov 2021
Dec 2021

LOGIC Show/hide trigger exists. Hidden unless: #37 Question "
Did you use the combined oral contraceptive pill for the entire pandemic (since 2020)?

" is one of the following answers ("No")

52. **Did you use any hormonal method or copper IUD after you stopped using combined oral contraceptives? Select which method you used next.** *If you started the same method again, select that method.*

- Combined oral contraceptive pill (e.g. Microgynon, Marvelon, Yasmin)
- Progestin only pill (e.g. Minipill)
- Contraceptive Injection (e.g. Depo-Provera, Sayana Press, Noristerat)
- Hormonal IUD/IUS/Coil (e.g. Mirena, Kyleena, Jaydess, Levosert or Skyla)
- Contraceptive patch (e.g. Ortho Evra, Xulane
- Vaginal ring (e.g. Nuvaring)
- Contraceptive Implant (e.g. Nexplanon)
- Copper IUD
- None of the above

LOGIC Show/hide trigger exists. Hidden unless: #38 Question "
Did you use the progestin only pill for the entire pandemic (since 2020)?

" is one of the following answers ("No")

53. **Did you use any hormonal method or copper IUD after you stopped using the progestin-only pill?** *Select which method you used next. If you started the same method again, select that method.*

- Combined oral contraceptive pill (e.g. Microgynon, Marvelon, Yasmin)
- Progestin only pill (e.g. Minipill)
- Contraceptive Injection (e.g. Depo-Provera, Sayana Press, Noristerat)
- Hormonal IUD/IUS/Coil (e.g. Mirena, Kyleena, Jaydess, Levosert or Skyla)
- Contraceptive patch (e.g. Ortho Evra, Xulane
- Vaginal ring (e.g. Nuvaring)
- Contraceptive Implant (e.g. Nexplanon)
- Copper IUD
- None of the above

LOGIC Show/hide trigger exists. Hidden unless: #39 Question "
Did you use the contraceptive injection for the entire pandemic (since 2020)?

" is one of the following answers ("No")

54. **Did you use any hormonal method or copper IUD after you stopped using the contraceptive injection?** *Select which method you used next. If you started the same method again, select that method.*

- Combined oral contraceptive pill (e.g. Microgynon, Marvelon, Yasmin)
- Progestin only pill (e.g. Minipill)
- Contraceptive Injection (e.g. Depo-Provera, Sayana Press, Noristerat)
- Hormonal IUD/IUS/Coil (e.g. Mirena, Kyleena, Jaydess, Levosert or Skyla)
- Contraceptive patch (e.g. Ortho Evra, Xulane
- Vaginal ring (e.g. Nuvaring)
- Contraceptive Implant (e.g. Nexplanon)
- Copper IUD
- None of the above

LOGIC Show/hide trigger exists. Hidden unless: #40 Question "
Did you use the hormonal IUD/IUS/Coil for the entire pandemic (since 2020)?

" is one of the following answers ("No")

55. **Did you use any hormonal method or copper IUD after you stopped using the hormonal IUD/IUS/Coil?** *Select which method you used next. If you started the same method again, select that method.*

- Combined oral contraceptive pill (e.g. Microgynon, Marvelon, Yasmin)
- Progestin only pill (e.g. Minipill)
- Contraceptive Injection (e.g. Depo-Provera, Sayana Press, Noristerat)
- Hormonal IUD/IUS/Coil (e.g. Mirena, Kyleena, Jaydess, Levosert or Skyla)
- Contraceptive patch (e.g. Ortho Evra, Xulane
- Vaginal ring (e.g. Nuvaring)
- Contraceptive Implant (e.g. Nexplanon)
- Copper IUD
- None of the above

Show/hide trigger exists. Hidden unless: #41 Question "
Did you use the contraceptive patch for the entire pandemic (since 2020)?

" is one of the following answers ("No")

56. **Did you use any hormonal method or copper IUD after you stopped using the contraceptive patch?** *Select which method you used next. If you started the same method again, select that method.*

- Combined oral contraceptive pill (e.g. Microgynon, Marvelon, Yasmin)
- Progestin only pill (e.g. Minipill)
- Contraceptive Injection (e.g. Depo-Provera, Sayana Press, Noristerat)
- Hormonal IUD/IUS/Coil (e.g. Mirena, Kyleena, Jaydess, Levosert or Skyla)
- Contraceptive patch (e.g. Ortho Evra, Xulane
- Vaginal ring (e.g. Nuvaring)
- Contraceptive Implant (e.g. Nexplanon)
- Copper IUD
- None of the above

Show/hide trigger exists. Hidden unless: #42 Question "
Did you use the vaginal ring for the entire pandemic (since 2020)?

" is one of the following answers ("No")

57. **Did you use any hormonal method or copper IUD after you stopped using the vaginal ring?** *Select which method you used next. If you started the same method again, select that method.*

- Combined oral contraceptive pill (e.g. Microgynon, Marvelon, Yasmin)
- Progestin only pill (e.g. Minipill)
- Contraceptive Injection (e.g. Depo-Provera, Sayana Press, Noristerat)
- Hormonal IUD/IUS/Coil (e.g. Mirena, Kyleena, Jaydess, Levosert or Skyla)
- Contraceptive patch (e.g. Ortho Evra, Xulane
- Vaginal ring (e.g. Nuvaring)
- Contraceptive Implant (e.g. Nexplanon)
- Copper IUD
- None of the above

LOGIC Show/hide trigger exists. Hidden unless: #43 Question "
Did you use the contraceptive implant for the entire pandemic (since 2020)?

" is one of the following answers ("No")

58. **Did you use any hormonal method or copper IUD after you stopped using the contraceptive implant?** *Select which method you used next. If you started the same method again, select that method.*

- Combined oral contraceptive pill (e.g. Microgynon, Marvelon, Yasmin)
- Progestin only pill (e.g. Minipill)
- Contraceptive Injection (e.g. Depo-Provera, Sayana Press, Noristerat)
- Hormonal IUD/IUS/Coil (e.g. Mirena, Kyleena, Jaydess, Levosert or Skyla)
- Contraceptive patch (e.g. Ortho Evra, Xulane
- Vaginal ring (e.g. Nuvaring)
- Contraceptive Implant (e.g. Nexplanon)
- Copper IUD
- None of the above

LOGIC Show/hide trigger exists. Hidden unless: #44 Question "
Did you use the copper IUD for the entire pandemic (since 2020)?

" is one of the following answers ("No")

59. **Did you use any hormonal method or copper IUD after you stopped using the copper IUD?** *Select which method you used next. If you started the same method again, select that method.*

- Combined oral contraceptive pill (e.g. Microgynon, Marvelon, Yasmin)
- Progestin only pill (e.g. Minipill)
- Contraceptive Injection (e.g. Depo-Provera, Sayana Press, Noristerat)
- Hormonal IUD/IUS/Coil (e.g. Mirena, Kyleena, Jaydess, Levosert or Skyla)
- Contraceptive patch (e.g. Ortho Evra, Xulane
- Vaginal ring (e.g. Nuvaring)
- Contraceptive Implant (e.g. Nexplanon)
- Copper IUD
- None of the above

LOGIC Hidden unless: #52 Question "

Did you use any hormonal method or copper IUD after you stopped using combined oral contraceptives? Select which method you used next. *If you started the same method again, select that method.*

" is one of the following answers ("Combined oral contraceptive pill (e.g. Microgynon, Marvelon, Yasmin)","Progestin only pill (e.g. Minipill)","Contraceptive Injection (e.g. Depo-Provera, Sayana Press, Noristerat)","Hormonal IUD/IUS/Coil (e.g. Mirena, Kyleena, Jaydess, Levosert or Skyla)","Contraceptive patch (e.g. Ortho Evra, Xulane","Vaginal ring (e.g. Nuvaring)","Contraceptive Implant (e.g. Nexplanon)","Copper IUD")

60. **When did you start using this method?**

- Feb 2020
- March 2020
- April 2020
- May 2020
- June 2020
- July 2020
- August 2020
- Sept 2020
- Oct 2020
- Nov 2020
- Dec 2020
- Jan 2021
- Feb 2021
- March 2021
- April 2021
- May 2021
- June 2021
- July 2021
- August 2021
- Sept 2021
- Oct 2021
- Nov 2021
- Dec 2021

LOGIC Hidden unless: #52 Question "

Did you use any hormonal method or copper IUD after you stopped using combined oral contraceptives? Select which method you used next. *If you started the same method again, select that method.*

" is one of the following answers ("Combined oral contraceptive pill (e.g. Microgynon, Marvelon, Yasmin)","Progestin only pill (e.g. Minipill)","Contraceptive Injection (e.g. Depo-Provera, Sayana Press, Noristerat)","Hormonal IUD/IUS/Coil (e.g. Mirena, Kyleena, Jaydess, Levosert or Skyla)","Contraceptive patch (e.g. Ortho Evra, Xulane","Vaginal ring (e.g. Nuvaring)","Contraceptive Implant (e.g. Nexplanon)","Copper IUD")

61. **Did you use this method for the rest of the pandemic (until now)?**

- ○ Yes
- ○ No

LOGIC Hidden unless: #53 Question "

Did you use any hormonal method or copper IUD after you stopped using the progestin-only pill? *Select which method you used next. If you started the same method again, select that method.*

" is one of the following answers ("Combined oral contraceptive pill (e.g. Microgynon, Marvelon, Yasmin)","Progestin only pill (e.g. Minipill)","Contraceptive Injection (e.g. Depo-Provera, Sayana Press, Noristerat)","Hormonal IUD/IUS/Coil (e.g. Mirena, Kyleena, Jaydess, Levosert or Skyla)","Contraceptive patch (e.g. Ortho Evra, Xulane","Vaginal ring (e.g. Nuvaring)","Contraceptive Implant (e.g. Nexplanon)","Copper IUD")

62. **When did you start using this method?**

- Feb 2020
- March 2020
- April 2020
- May 2020
- June 2020
- July 2020
- August 2020
- Sept 2020
- Oct 2020
- Nov 2020
- Dec 2020
- Jan 2021
- Feb 2021
- March 2021
- April 2021
- May 2021
- June 2021
- July 2021
- August 2021
- Sept 2021
- Oct 2021
- Nov 2021
- Dec 2021

LOGIC Hidden unless: #53 Question "

Did you use any hormonal method or copper IUD after you stopped using the progestin-only pill? *Select which method you used next. If you started the same method again, select that method.*

" is one of the following answers ("Combined oral contraceptive pill (e.g. Microgynon, Marvelon, Yasmin)","Progestin only pill (e.g. Minipill)","Contraceptive Injection (e.g. Depo-Provera, Sayana Press, Noristerat)","Hormonal IUD/IUS/Coil (e.g. Mirena, Kyleena, Jaydess, Levosert or Skyla)","Contraceptive patch (e.g. Ortho Evra, Xulane","Vaginal ring (e.g. Nuvaring)","Contraceptive Implant (e.g. Nexplanon)","Copper IUD")

63. **Did you use this method for the rest of the pandemic (until now)?**

- ○ Yes
- ○ No

LOGIC Hidden unless: #54 Question "
Did you use any hormonal method or copper IUD after you stopped using the contraceptive injection? *Select which method you used next. If you started the same method again, select that method.*

" is one of the following answers ("Combined oral contraceptive pill (e.g. Microgynon, Marvelon, Yasmin)","Progestin only pill (e.g. Minipill)","Contraceptive Injection (e.g. Depo-Provera, Sayana Press, Noristerat)","Hormonal IUD/IUS/Coil (e.g. Mirena, Kyleena, Jaydess, Levosert or Skyla)","Contraceptive patch (e.g. Ortho Evra, Xulane","Vaginal ring (e.g. Nuvaring)","Contraceptive Implant (e.g. Nexplanon)","Copper IUD")

64. **When did you start using this method?**

Feb 2020
March 2020
April 2020
May 2020
June 2020
July 2020
August 2020
Sept 2020
Oct 2020
Nov 2020
Dec 2020
Jan 2021
Feb 2021
March 2021
April 2021
May 2021
June 2021
July 2021
August 2021
Sept 2021
Oct 2021
Nov 2021
Dec 2021

LOGIC Hidden unless: #55 Question "
Did you use any hormonal method or copper IUD after you stopped using the hormonal IUD/IUS/Coil? *Select which method you used next. If you started the same method again, select that method.*

" is one of the following answers ("Combined oral contraceptive pill (e.g. Microgynon, Marvelon, Yasmin)","Progestin only pill (e.g. Minipill)","Contraceptive Injection (e.g. Depo-Provera, Sayana Press, Noristerat)","Hormonal IUD/IUS/Coil (e.g. Mirena, Kyleena, Jaydess, Levosert or Skyla)","Contraceptive patch (e.g. Ortho Evra, Xulane","Vaginal ring (e.g. Nuvaring)","Contraceptive Implant (e.g. Nexplanon)","Copper IUD")

65. **Did you use this method for the rest of the pandemic (until now)?**

- Yes
- No

LOGIC Hidden unless: #55 Question "

Did you use any hormonal method or copper IUD after you stopped using the hormonal IUD/IUS/Coil? *Select which method you used next. If you started the same method again, select that method.*

" is one of the following answers ("Combined oral contraceptive pill (e.g. Microgynon, Marvelon, Yasmin)","Progestin only pill (e.g. Minipill)","Contraceptive Injection (e.g. Depo-Provera, Sayana Press, Noristerat)","Hormonal IUD/IUS/Coil (e.g. Mirena, Kyleena, Jaydess, Levosert or Skyla)","Contraceptive patch (e.g. Ortho Evra, Xulane","Vaginal ring (e.g. Nuvaring)","Contraceptive Implant (e.g. Nexplanon)","Copper IUD")

66. **When did you start using this method?**

- Feb 2020
- March 2020
- April 2020
- May 2020
- June 2020
- July 2020
- August 2020
- Sept 2020
- Oct 2020
- Nov 2020
- Dec 2020
- Jan 2021
- Feb 2021
- March 2021
- April 2021
- May 2021
- June 2021
- July 2021
- August 2021
- Sept 2021
- Oct 2021
- Nov 2021
- Dec 2021

LOGIC Hidden unless: #55 Question "

Did you use any hormonal method or copper IUD after you stopped using the hormonal IUD/IUS/Coil? *Select which method you used next. If you started the same method again, select that method.*

" is one of the following answers ("Combined oral contraceptive pill (e.g. Microgynon, Marvelon, Yasmin)","Progestin only pill (e.g. Minipill)","Contraceptive Injection (e.g. Depo-Provera, Sayana Press, Noristerat)","Hormonal IUD/IUS/Coil (e.g. Mirena, Kyleena, Jaydess, Levosert or Skyla)","Contraceptive patch (e.g. Ortho Evra, Xulane","Vaginal ring (e.g. Nuvaring)","Contraceptive Implant (e.g. Nexplanon)","Copper IUD")

67. **Did you use this method for the rest of the pandemic (until now)?**

- Yes
- No

LOGIC Hidden unless: #56 Question "

Did you use any hormonal method or copper IUD after you stopped using the contraceptive patch? *Select which method you used next. If you started the same method again, select that method.*

" is one of the following answers ("Combined oral contraceptive pill (e.g. Microgynon, Marvelon, Yasmin)","Progestin only pill (e.g. Minipill)","Contraceptive Injection (e.g. Depo-Provera, Sayana Press, Noristerat)","Hormonal IUD/IUS/Coil (e.g. Mirena, Kyleena, Jaydess, Levosert or Skyla)","Contraceptive patch (e.g. Ortho Evra, Xulane","Vaginal ring (e.g. Nuvaring)","Contraceptive Implant (e.g. Nexplanon)","Copper IUD")

68. **When did you start using this method?**

Feb 2020
March 2020
April 2020
May 2020
June 2020
July 2020
August 2020
Sept 2020
Oct 2020
Nov 2020
Dec 2020
Jan 2021
Feb 2021
March 2021
April 2021
May 2021
June 2021
July 2021
August 2021
Sept 2021
Oct 2021
Nov 2021
Dec 2021

LOGIC Hidden unless: #56 Question "

Did you use any hormonal method or copper IUD after you stopped using the contraceptive patch? *Select which method you used next. If you started the same method again, select that method.*

" is one of the following answers ("Combined oral contraceptive pill (e.g. Microgynon, Marvelon, Yasmin)","Progestin only pill (e.g. Minipill)","Contraceptive Injection (e.g. Depo-Provera, Sayana Press, Noristerat)","Hormonal IUD/IUS/Coil (e.g. Mirena, Kyleena, Jaydess, Levosert or Skyla)","Contraceptive patch (e.g. Ortho Evra, Xulane","Vaginal ring (e.g. Nuvaring)","Contraceptive Implant (e.g. Nexplanon)","Copper IUD")

69. **Did you use this method for the rest of the pandemic (until now)?**

- Yes
- No

Hidden unless: #57 Question "

Did you use any hormonal method or copper IUD after you stopped using the vaginal ring? *Select which method you used next. If you started the same method again, select that method.*

" is one of the following answers ("Combined oral contraceptive pill (e.g. Microgynon, Marvelon, Yasmin)","Progestin only pill (e.g. Minipill)","Contraceptive Injection (e.g. Depo-Provera, Sayana Press, Noristerat)","Hormonal IUD/IUS/Coil (e.g. Mirena, Kyleena, Jaydess, Levosert or Skyla)","Contraceptive patch (e.g. Ortho Evra, Xulane","Vaginal ring (e.g. Nuvaring)","Contraceptive Implant (e.g. Nexplanon)","Copper IUD")

70. **When did you start using this method?**

- Feb 2020
- March 2020
- April 2020
- May 2020
- June 2020
- July 2020
- August 2020
- Sept 2020
- Oct 2020
- Nov 2020
- Dec 2020
- Jan 2021
- Feb 2021
- March 2021
- April 2021
- May 2021
- June 2021
- July 2021
- August 2021
- Sept 2021
- Oct 2021
- Nov 2021
- Dec 2021

Hidden unless: #57 Question "

Did you use any hormonal method or copper IUD after you stopped using the vaginal ring? *Select which method you used next. If you started the same method again, select that method.*

" is one of the following answers ("Combined oral contraceptive pill (e.g. Microgynon, Marvelon, Yasmin)","Progestin only pill (e.g. Minipill)","Contraceptive Injection (e.g. Depo-Provera, Sayana Press, Noristerat)","Hormonal IUD/IUS/Coil (e.g. Mirena, Kyleena, Jaydess, Levosert or Skyla)","Contraceptive patch (e.g. Ortho Evra, Xulane","Vaginal ring (e.g. Nuvaring)","Contraceptive Implant (e.g. Nexplanon)","Copper IUD")

71. **Did you use this method for the rest of the pandemic (until now)?**

- Yes
- No

LOGIC Hidden unless: #58 Question "
Did you use any hormonal method or copper IUD after you stopped using the contraceptive implant? *Select which method you used next. If you started the same method again, select that method.*

" is one of the following answers ("Combined oral contraceptive pill (e.g. Microgynon, Marvelon, Yasmin)","Progestin only pill (e.g. Minipill)","Contraceptive Injection (e.g. Depo-Provera, Sayana Press, Noristerat)","Hormonal IUD/IUS/Coil (e.g. Mirena, Kyleena, Jaydess, Levosert or Skyla)","Contraceptive patch (e.g. Ortho Evra, Xulane","Vaginal ring (e.g. Nuvaring)","Contraceptive Implant (e.g. Nexplanon)","Copper IUD")

72. **When did you start using this method?**

Feb 2020
March 2020
April 2020
May 2020
June 2020
July 2020
August 2020
Sept 2020
Oct 2020
Nov 2020
Dec 2020
Jan 2021
Feb 2021
March 2021
April 2021
May 2021
June 2021
July 2021
August 2021
Sept 2021
Oct 2021
Nov 2021
Dec 2021

LOGIC Hidden unless: #58 Question "
Did you use any hormonal method or copper IUD after you stopped using the contraceptive implant? *Select which method you used next. If you started the same method again, select that method.*

" is one of the following answers ("Combined oral contraceptive pill (e.g. Microgynon, Marvelon, Yasmin)","Progestin only pill (e.g. Minipill)","Contraceptive Injection (e.g. Depo-Provera, Sayana Press, Noristerat)","Hormonal IUD/IUS/Coil (e.g. Mirena, Kyleena, Jaydess, Levosert or Skyla)","Contraceptive patch (e.g. Ortho Evra, Xulane","Vaginal ring (e.g. Nuvaring)","Contraceptive Implant (e.g. Nexplanon)","Copper IUD")

73. **Did you use this method for the rest of the pandemic (until now)?**

- Yes
- No

LOGIC Hidden unless: #59 Question "

Did you use any hormonal method or copper IUD after you stopped using the copper IUD? *Select which method you used next. If you started the same method again, select that method.*

" is one of the following answers ("Combined oral contraceptive pill (e.g. Microgynon, Marvelon, Yasmin)","Progestin only pill (e.g. Minipill)","Contraceptive Injection (e.g. Depo-Provera, Sayana Press, Noristerat)","Hormonal IUD/IUS/Coil (e.g. Mirena, Kyleena, Jaydess, Levosert or Skyla)","Contraceptive patch (e.g. Ortho Evra, Xulane","Vaginal ring (e.g. Nuvaring)","Contraceptive Implant (e.g. Nexplanon)","Copper IUD")

74. **When did you start using this method?**

- Feb 2020
- March 2020
- April 2020
- May 2020
- June 2020
- July 2020
- August 2020
- Sept 2020
- Oct 2020
- Nov 2020
- Dec 2020
- Jan 2021
- Feb 2021
- March 2021
- April 2021
- May 2021
- June 2021
- July 2021
- August 2021
- Sept 2021
- Oct 2021
- Nov 2021
- Dec 2021

LOGIC Hidden unless: #59 Question "

Did you use any hormonal method or copper IUD after you stopped using the copper IUD? *Select which method you used next. If you started the same method again, select that method.*

" is one of the following answers ("Combined oral contraceptive pill (e.g. Microgynon, Marvelon, Yasmin)","Progestin only pill (e.g. Minipill)","Contraceptive Injection (e.g. Depo-Provera, Sayana Press, Noristerat)","Hormonal IUD/IUS/Coil (e.g. Mirena, Kyleena, Jaydess, Levosert or Skyla)","Contraceptive patch (e.g. Ortho Evra, Xulane","Vaginal ring (e.g. Nuvaring)","Contraceptive Implant (e.g. Nexplanon)","Copper IUD")

75. **Did you use this method for the rest of the pandemic (until now)?**

- Yes
- No

LOGIC Show/hide trigger exists. Hidden unless: #35 Question "**Were you using a hormonal contraceptive or a copper IUDin January 2020?** " is one of the following answers ("No")

76. **Have you used any hormonal contraceptives or a copper IUD since January 2020?** Select the first method you used after January 2020.

- ○ Combined oral contraceptive pill (e.g. Microgynon, Marvelon, Yasmin)
- ○ Progestin only pill (e.g. Minipill)
- ○ Contraceptive Injection (e.g. Depo-Provera, Sayana Press, Noristerat)
- ○ Hormonal IUD/IUS/Coil (e.g. Mirena, Kyleena, Jaydess, Levosert or Skyla)
- ○ Contraceptive patch (e.g. Ortho Evra, Xulane
- ○ Vaginal ring (e.g. Nuvaring)
- ○ Contraceptive Implant (e.g. Nexplanon)
- ○ Copper IUD
- ○ I have not used a hormonal contraceptive or copper IUD since January 2020

LOGIC Hidden unless: #76 Question "

Have you used any hormonal contraceptives or a copper IUD since January 2020? Select the first method you used after January 2020.

" is one of the following answers ("Combined oral contraceptive pill (e.g. Microgynon, Marvelon, Yasmin)")

77. **When did you start using the combined oral contraceptive pill?**

Jan 2020
Feb 2020
March 2020
April 2020
May 2020
June 2020
July 2020
August 2020
Sept 2020
Oct 2020
Nov 2020
Dec 2020
Jan 2021
Feb 2021
March 2021
April 2021
May 2021
June 2021
July 2021
August 2021
Sept 2021
Oct 2021
Nov 2021
Dec 2021

LOGIC Hidden unless: #76 Question "
Have you used any hormonal contraceptives or a copper IUD since January 2020? Select the first method you used after January 2020.

" is one of the following answers ("Combined oral contraceptive pill (e.g. Microgynon, Marvelon, Yasmin)")

78. **Have you used combined oral contraceptives for the rest of the pandemic (until now)?**

- Yes
- No

LOGIC Hidden unless: #76 Question "
Have you used any hormonal contraceptives or a copper IUD since January 2020? Select the first method you used after January 2020.

" is one of the following answers ("Progestin only pill (e.g. Minipill)")

79. **When did you start using the progestin-only pill?**

- Jan 2020
- Feb 2020
- March 2020
- April 2020
- May 2020
- June 2020
- July 2020
- August 2020
- Sept 2020
- Oct 2020
- Nov 2020
- Dec 2020
- Jan 2021
- Feb 2021
- March 2021
- April 2021
- May 2021
- June 2021
- July 2021
- August 2021
- Sept 2021
- Oct 2021
- Nov 2021
- Dec 2021

LOGIC Hidden unless: #76 Question "
Have you used any hormonal contraceptives or a copper IUD since January 2020? Select the first method you used after January 2020.

" is one of the following answers ("Progestin only pill (e.g. Minipill)")

80. **Have you used the progestin-only pill for the rest of the pandemic (until now)?**

- Yes
- No

Hidden unless: #76 Question "
Have you used any hormonal contraceptives or a copper IUD since January 2020? Select the first method you used after January 2020.

" is one of the following answers ("Contraceptive Injection (e.g. Depo-Provera, Sayana Press, Noristerat)")

81. **When did you start using the contraceptive injection?**

- Jan 2020
- Feb 2020
- March 2020
- April 2020
- May 2020
- June 2020
- July 2020
- August 2020
- Sept 2020
- Oct 2020
- Nov 2020
- Dec 2020
- Jan 2021
- Feb 2021
- March 2021
- April 2021
- May 2021
- June 2021
- July 2021
- August 2021
- Sept 2021
- Oct 2021
- Nov 2021
- Dec 2021

Hidden unless: #76 Question "
Have you used any hormonal contraceptives or a copper IUD since January 2020? Select the first method you used after January 2020.

" is one of the following answers ("Contraceptive Injection (e.g. Depo-Provera, Sayana Press, Noristerat)")

82. **Have you used the contraceptive injection for the rest of the pandemic (until now)?**

- Yes
- No

LOGIC Hidden unless: #76 Question "
Have you used any hormonal contraceptives or a copper IUD since January 2020? Select the first method you used after January 2020.

" is one of the following answers ("Hormonal IUD/IUS/Coil (e.g. Mirena, Kyleena, Jaydess, Levosert or Skyla)")

83. **When did you start using the hormonal IUS/IUD/Coil?**

- Jan 2020
- Feb 2020
- March 2020
- April 2020
- May 2020
- June 2020
- July 2020
- August 2020
- Sept 2020
- Oct 2020
- Nov 2020
- Dec 2020
- Jan 2021
- Feb 2021
- March 2021
- April 2021
- May 2021
- June 2021
- July 2021
- August 2021
- Sept 2021
- Oct 2021
- Nov 2021
- Dec 2021

LOGIC Hidden unless: #76 Question "
Have you used any hormonal contraceptives or a copper IUD since January 2020? Select the first method you used after January 2020.

" is one of the following answers ("Hormonal IUD/IUS/Coil (e.g. Mirena, Kyleena, Jaydess, Levosert or Skyla)")

84. **Have you used the hormonal IUD/IUS/Coil for the rest of the pandemic (until now)?**

- Yes
- No

LOGIC Hidden unless: #76 Question "

Have you used any hormonal contraceptives or a copper IUD since January 2020? Select the first method you used after January 2020.

" is one of the following answers ("Contraceptive patch (e.g. Ortho Evra, Xulane")

85. **When did you start using the contraceptive patch?**

- Jan 2020
- Feb 2020
- March 2020
- April 2020
- May 2020
- June 2020
- July 2020
- August 2020
- Sept 2020
- Oct 2020
- Nov 2020
- Dec 2020
- Jan 2021
- Feb 2021
- March 2021
- April 2021
- May 2021
- June 2021
- July 2021
- August 2021
- Sept 2021
- Oct 2021
- Nov 2021
- Dec 2021

LOGIC Hidden unless: #76 Question "

Have you used any hormonal contraceptives or a copper IUD since January 2020? Select the first method you used after January 2020.

" is one of the following answers ("Contraceptive patch (e.g. Ortho Evra, Xulane")

86. **Have you used the contraceptive patch for the rest of the pandemic (until now)?**

- Yes
- No

LOGIC Hidden unless: #76 Question "

Have you used any hormonal contraceptives or a copper IUD since January 2020? Select the first method you used after January 2020.

" is one of the following answers ("Vaginal ring (e.g. Nuvaring)")

87. **When did you start using the vaginal ring?**

- Jan 2020
- Feb 2020
- March 2020
- April 2020
- May 2020
- June 2020
- July 2020
- August 2020
- Sept 2020
- Oct 2020
- Nov 2020
- Dec 2020
- Jan 2021
- Feb 2021
- March 2021
- April 2021
- May 2021
- June 2021
- July 2021
- August 2021
- Sept 2021
- Oct 2021
- Nov 2021
- Dec 2021

LOGIC Hidden unless: #76 Question "

Have you used any hormonal contraceptives or a copper IUD since January 2020? Select the first method you used after January 2020.

" is one of the following answers ("Vaginal ring (e.g. Nuvaring)")

88. **Have you used the vaginal ring for the rest of the pandemic (until now)?**

- Yes
- No

LOGIC Hidden unless: #76 Question "
Have you used any hormonal contraceptives or a copper IUD since January 2020? Select the first method you used after January 2020.

" is one of the following answers ("Contraceptive Implant (e.g. Nexplanon)")

89. **When did you start using the contraceptive implant?**

- Jan 2020
- Feb 2020
- March 2020
- April 2020
- May 2020
- June 2020
- July 2020
- August 2020
- Sept 2020
- Oct 2020
- Nov 2020
- Dec 2020
- Jan 2021
- Feb 2021
- March 2021
- April 2021
- May 2021
- June 2021
- July 2021
- August 2021
- Sept 2021
- Oct 2021
- Nov 2021
- Dec 2021

LOGIC Hidden unless: #76 Question "
Have you used any hormonal contraceptives or a copper IUD since January 2020? Select the first method you used after January 2020.

" is one of the following answers ("Contraceptive Implant (e.g. Nexplanon)")

90. **Have you used the contraceptive implant for the rest of the pandemic (until now)?**

- Yes
- No

LOGIC Hidden unless: #76 Question "

Have you used any hormonal contraceptives or a copper IUD since January 2020? Select the first method you used after January 2020.

" is one of the following answers ("Copper IUD")

91. **When did you start using the copper IUD?**

- Jan 2020
- Feb 2020
- March 2020
- April 2020
- May 2020
- June 2020
- July 2020
- August 2020
- Sept 2020
- Oct 2020
- Nov 2020
- Dec 2020
- Jan 2021
- Feb 2021
- March 2021
- April 2021
- May 2021
- June 2021
- July 2021
- August 2021
- Sept 2021
- Oct 2021
- Nov 2021
- Dec 2021

LOGIC Hidden unless: #76 Question "

Have you used any hormonal contraceptives or a copper IUD since January 2020? Select the first method you used after January 2020.

" is one of the following answers ("Copper IUD")

92. **Have you used the copper IUD for the rest of the pandemic (until now)?**

- Yes
- No

LOGIC Show/hide trigger exists.

93. **Did you undergo any gynecologic surgeries/procedures since the beginning of 2020?**

- Yes
- No

LOGIC Hidden unless: #93 Question "**Did you undergo any gynecologic surgeries/procedures since the beginning of 2020?** is one of the following answers ("Yes")

94. **What were the surgical procedures you underwent?** (check all that apply)

- ☐ Endometrial biopsy (a small piece of the uterus is removed)
- ☐ Myomectomy (fibroid removal surgery)
- ☐ Dilation and curettage (surgical miscarriage or abortion)
- ☐ Polypectomy (removal of polyp)
- ☐ Hysteroscopy (removal of uterus)
- ☐ Loop electrosurgical excision procedure (LEEP, to remove precancerous cells from the cervix)
- ☐ None of the above

LOGIC Hidden unless: #93 Question "**Did you undergo any gynecologic surgeries/procedures since the beginning of 2020?** is one of the following answers ("Yes")

95. **When was your gynecologic procedure?** (If you have undergone several procedures, select the date of the first one)

Before 2020
Jan 2020
Feb 2020
Mar 2020
Apr 2020
May 2020
June 2020
Jul 2020
Aug 2020
Sept 2020
Oct 2020
Nov 2020
Dec 2020
Jan 2021
Feb 2021
Mar 2021
Apr 2021
May 2021
June 2021
July 2021
August 2021
September 2021
October 2021
Nov 2021
Dec 2021

96. **Has your weight changed since the beginning of 2020?**

- ○ Yes, I have gained weight
- ○ Yes, I have lost weight
- ○ Yes, I lost and gained weight at different times
- ○ No, I am about the same weight
- ○ Prefer not to say

97. **Which of the following have you experienced during the pandemic?** (check all that apply)

- ☐ Loss of loved ones (e.g. bereavement)
- ☐ A close family member or friend had to be hospitalized because of serious illness or injury
- ☐ Serious illness or injury requiring my hospitalization
- ☐ Increased stigma or discrimination from other people
- ☐ Personal financial loss
- ☐ Not having enough basic supplies (e.g., food, water, medications, a place to stay)
- ☐ Anxiety / depression
- ☐ Changes to my typical sleep pattern
- ☐ Increased alcohol or other substance use
- ☐ Loneliness
- ☐ Fear
- ☐ Worry for friends and family
- ☐ None of the above

98. **In total, how much time have you spent in isolation or quarantine because of lockdowns or travel measures, and/or following possible COVID-19 exposure or positive COVID-19 test(s)?**

- ○ None
- ○ Less than a week
- ○ 1-4 weeks
- ○ 5-8 weeks
- ○ 9-12 weeks
- ○ More than 12 weeks

99. **Did you have to balance working from home and taking care of others (e.g., parents, kids, partners) during the pandemic?**

- ○ Yes
- ○ No

100. **How comfortable was your home/living space situation during the pandemic?** (in terms of space available for yourself)

- ○ Very uncomfortable
- ○ Slightly uncomfortable
- ○ Neither comfortable / uncomfortable
- ○ Slightly comfortable
- ○ Very comfortable

101. When you were most restricted due to the pandemic, did you have access to any of the following outdoor spaces from your home?

- ☐ Private or shared garden / yard
- ☐ Balcony / terrace
- ☐ Other accessible spaces (e.g. park nearby)
- ☐ None of the above

102. **On a scale from 0-10, how stressed have you been feeling, on average, since the beginning of the pandemic?**

Not stressed ——— Extremely stressed

LOGIC Show/hide trigger exists.

103. **Have you ever been infected with COVID-19?** *

- ○ Yes
- ○ No
- ○ I'm not sure
- ○ Prefer not to answer

Show/hide trigger exists. Hidden unless: #103 Question "
Have you ever been infected with COVID-19?

" is one of the following answers ("Yes")

104. **Did you experience any symptoms with COVID-19?**

- I did not experience symptoms
- I had some mild cold symptoms but they didn’t interfere with my activities
- I had moderate symptoms that interfered with my activities
- I had moderate symptoms that prevented me from my activities
- I had severe symptoms and I had to get emergency care but I did not need to stay in the hospital
- I had severe symptoms and I needed oxygen therapy in the hospital but I did not need a ventilator
- I had severe symptoms and I needed a ventilator

Hidden unless: #104 Question "**Did you experience any symptoms with COVID-19?**" is one of the following answers ("I had some mild cold symptoms but they didn’t interfere with my activities","I had moderate symptoms that interfered with my activities","I had moderate symptoms that prevented me from my activities","I had severe symptoms and I had to get emergency care but I did not need to stay in the hospital","I had severe symptoms and I needed oxygen therapy in the hospital but I did not need a ventilator","I had severe symptoms and I needed a ventilator")

105. **When did your first symptoms start?**

I don't know
January 2020
February 2020
March 2020
April 2020
May 2020
June 2020
July 2020
August 2020
September 2020
October 2020
November 2020
December 2020
January 2021
February 2021
March 2021
April 2021
May 2021
June 2021
July 2021
August 2021
September 2021
October 2021
November 2021
December 2021

LOGIC Show/hide trigger exists. Hidden unless: #103 Question "
Have you ever been infected with COVID-19?

" is one of the following answers ("Yes")

106. **Have you had at least one positive test for COVID-19?** *

- Yes
- No but I have had a clinical diagnosis by a doctor based on symptoms
- No

LOGIC Hidden unless: #106 Question "
Have you had at least one positive test for COVID-19?

" is one of the following answers ("Yes")

107. **When was your first positive test?**

I don't know
January 2020
February 2020
March 2020
April 2020
May 2020
June 2020
July 2020
August 2020
September 2020
October 2020
November 2020
December 2020
January 2021
February 2021
March 2021
April 2021
May 2021
June 2021
July 2021
August 2021
September 2021
October 2021
November 2021
December 2021

LOGIC Hidden unless: #106 Question "
Have you had at least one positive test for COVID-19?

" is one of the following answers ("No but I have had a clinical diagnosis by a doctor based on symptoms")

108. **When were you diagnosed?**

- I don't know
- January 2020
- February 2020
- March 2020
- April 2020
- May 2020
- June 2020
- July 2020
- August 2020
- September 2020
- October 2020
- November 2020
- December 2020
- January 2021
- February 2021
- March 2021
- April 2021
- May 2021
- June 2021
- July 2021
- August 2021
- September 2021
- October 2021
- November 2021
- December 2021

LOGIC Hidden unless: #106 Question "
Have you had at least one positive test for COVID-19?

" is one of the following answers ("Yes")

109. **What was the type of the test that came back positive?**

- PCR test (nose/throat swab)
- Antigen / Rapid test
- I don't know which type of test but it was a nose/throat swab
- Antibody blood test
- Other (please specify)

LOGIC Show/hide trigger exists. Hidden unless: #103 Question "
Have you ever been infected with COVID-19?
" is one of the following answers ("Yes")

110. **Have you experienced long-haul COVID-19 (symptoms lasting more 1 month)?** *

- Yes
- No
- I'm not sure
- Prefer not to answer

LOGIC Hidden unless: #110 Question "
Have you experienced long-haul COVID-19 (symptoms lasting more 1 month)?
" is one of the following answers ("Yes")

111. **Are you still experiencing symptoms from your COVID-19 infection?**

- Yes
- No

LOGIC Hidden unless: #110 Question "
Have you experienced long-haul COVID-19 (symptoms lasting more 1 month)?
" is one of the following answers ("Yes")

112. **How would you rate how you feel today, on a scale of 0-100% (with 100% being how you felt before COVID-19)?**

1 ———————— 100

LOGIC Hidden unless: #103 Question "
Have you ever been infected with COVID-19?
" is one of the following answers ("Yes")

113. **Which of the following symptoms did you experience from your COVID-19 infection (list 1)?** (check all that apply)

- Brain fog / cognitive dysfunction
- Memory impairment
- Speech and language symptoms (e.g. difficulty finding words)
- Sensorimotor symptoms (e.g. tingling/pins and needles)
- Dizziness and balance issues
- Change in smell and taste
- None of the above

Hidden unless: #103 Question "
Have you ever been infected with COVID-19?

" is one of the following answers ("Yes")

114. **Which of the following symptoms did you experience from your COVID-19 infection (list 2)?** (check all that apply)

- ☐ Insomnia (unable to sleep)
- ☐ Headache
- ☐ Disturbed sleep
- ☐ Fatigue
- ☐ Exhaustion after exercise/effort
- ☐ Chills, flushing, sweats
- ☐ None of the above

Hidden unless: #103 Question "
Have you ever been infected with COVID-19?

" is one of the following answers ("Yes")

115. **Which of the following symptoms did you experience from your COVID-19 infection (list 3)?** (check all that apply)

- ☐ Fever (>98.6F, >37C)
- ☐ Heart palpitations (extra awareness of heart beat or irregular heart beat)
- ☐ Tachycardia (heart beating too fast)
- ☐ Pain/burning in the chest
- ☐ Chest tightness
- ☐ Muscle aches
- ☐ None of the above

LOGIC Hidden unless: #103 Question "
Have you ever been infected with COVID-19?

" is one of the following answers ("Yes")

116. **Which of the following symptoms did you experience from your COVID-19 infection (list 4)?** (check all that apply)

- ☐ Joint pain
- ☐ Sore throat
- ☐ Vision symptoms (e.g. blurred vision, flashing)
- ☐ Ringing in the ears
- ☐ Shortness of breath
- ☐ Dry cough
- ☐ None of the above

LOGIC Hidden unless: #103 Question "
Have you ever been infected with COVID-19?

" is one of the following answers ("Yes")

117. **Which of the following symptoms did you experience from your COVID-19 infection (list 5)?** (check all that apply)

- ☐ Breathing difficulty
- ☐ Diarrhea
- ☐ Loss of appetite
- ☐ Nausea
- ☐ Abdominal pain
- ☐ None of the above

Page exit logic: Skip / Disqualify Logic
IF: #1 Question "
Please confirm that you have read the information above and agree with the following statements:

" is not one of the following answers ("I have read the information and voluntarily agree to participate in this study","I am 18 years of age or older","I experience menstrual periods (or withdrawal bleeds)","I have not been pregnant or breastfeeding since January 2020") **THEN:** Jump to page 9 - Thank You!

LOGIC Show/hide trigger exists.

118. **Have you been vaccinated against COVID-19?**

- ○ No
- ○ Yes, one shot
- ○ Yes, two shots
- ○ Yes, three shots
- ○ I am not sure because I am currently enrolled in a COVID-19 clinical trial

LOGIC Hidden unless: #118 Question "
Have you been vaccinated against COVID-19?
" is one of the following answers ("Yes, one shot","Yes, two shots","Yes, three shots")

119. **Which type of vaccine did you have for your first dose?**

- ○ Pfizer-BioNTech
- ○ Johnson & Johnson
- ○ Moderna
- ○ Oxford-AstraZeneca
- ○ Sinopharm
- ○ I am not sure
- ○ Other - Write In

LOGIC Show/hide trigger exists. Hidden unless: #118 Question "
Have you been vaccinated against COVID-19?
" is one of the following answers ("Yes, one shot","Yes, two shots","Yes, three shots")

120. **When did you get your first dose?**

- Before 2021
- January 2021
- February 2021
- March 2021
- April 2021
- May 2021
- June 2021
- July 2021
- August 2021
- September 2021
- October 2021
- November 2021
- December 2021
- I don't know

LOGIC Show/hide trigger exists. Hidden unless: #120 Question "**When did you get your first dose?**" is one of the following answers ("January 2021","February 2021","March 2021","April 2021","May 2021","June 2021","July 2021","August 2021","September 2021")

121. **What day of the month were you vaccinated?**

- I don't know
- 1
- 2
- 3
- 4
- 5
- 6
- 7
- 8
- 9
- 10
- 11
- 12
- 13
- 14
- 15
- 16
- 17
- 18
- 19
- 20
- 21
- 22
- 23
- 24
- 25
- 26
- 27
- 28
- 29
- 30
- 31

LOGIC Hidden unless: #121 Question "**What day of the month were you vaccinated?**" is one of the following answers ("1","2","3","4","5","6","7","8","9","10","11","12","13","14","15","16","17","18","19","20","21","22","23","24","25","26","27","28","29","30","31")

122. **How certain are you of your vaccination date?**

- Very certain
- Certain +/- 3 days
- Not certain

Show/hide trigger exists. Hidden unless: #118 Question "
Have you been vaccinated against COVID-19?

" is one of the following answers ("Yes, one shot","Yes, two shots","Yes, three shots")

123. **What was your overall experience with your first vaccine dose?**

- ○ No side-effects
- ○ I had some mild symptoms but they didn't interfere with my activities
- ○ I had symptoms that interfered with my activities
- ○ I had symptoms that prevented me from my activities
- ○ I had to get emergency care or go to hospital for my symptoms

Show/hide trigger exists. Hidden unless: #123 Question "**What was your overall experience with your first vaccine dose?**" is one of the following answers ("I had some mild symptoms but they didn't interfere with my activities","I had symptoms that interfered with my activities","I had symptoms that prevented me from my activities","I had to get emergency care or go to hospital for my symptoms")

124. **Which symptoms did you experience from your first vaccine dose?** (check all that apply)

- ☐ Changes in periods / cycles
- ☐ Pain at the injection site
- ☐ Chills / Fever
- ☐ Dry cough
- ☐ Tiredness
- ☐ Aches and pains
- ☐ Sore throat
- ☐ Diarrhea
- ☐ Conjunctivitis (pink eye)
- ☐ Headache
- ☐ Loss of taste or smell
- ☐ A rash on skin, or discolouration of fingers or toes
- ☐ Difficulty breathing or shortness of breath
- ☐ Chest pain or pressure
- ☐ Loss of speech or movement
- ☐ Other (please specify)

Hidden unless: #124 Question "**Which symptoms did you experience from your first vaccine dose?** (check all that apply)" is one of the following answers ("Changes in periods / cycles")

125. **Which of the following menstrual changes did you experience?** (check all that apply)

Bleeding

- ☐ Heavier
- ☐ Lighter
- ☐ Spotting

Cycles (length of time between periods)

- ☐ Shorter
- ☐ Longer
- ☐ Become irregular

Period (bleeding days)

- ☐ Longer
- ☐ Shorter
- ☐ Skip / Stopped

Other

- ☐ Other (please specify)

Hidden unless: #118 Question "

Have you been vaccinated against COVID-19?

" is one of the following answers ("Yes, two shots","Yes, three shots")

126. **Which type of vaccine did you have for your second dose?**

- ○ Pfizer-BioNTech
- ○ Johnson & Johnson
- ○ Moderna
- ○ Oxford-AstraZeneca
- ○ Sinopharm
- ○ Other (please specify)
- ○ I am not sure

LOGIC Show/hide trigger exists. Hidden unless: #118 Question "
Have you been vaccinated against COVID-19?

" is one of the following answers ("Yes, two shots","Yes, three shots")

127. **When did you get your second dose?**

- Before 2021
- January 2021
- February 2021
- March 2021
- April 2021
- May 2021
- June 2021
- July 2021
- August 2021
- September 2021
- October 2021
- November 2021
- December 2021
- I don't know

LOGIC Show/hide trigger exists. Hidden unless: #127 Question "**When did you get your second dose?**" is one of the following answers ("January 2021","February 2021","March 2021","April 2021","May 2021","June 2021","July 2021","August 2021","September 2021")

128. **What day of the month were you vaccinated?**

- I don't know
- 1
- 2
- 3
- 4
- 5
- 6
- 7
- 8
- 9
- 10
- 11
- 12
- 13
- 14
- 15
- 16
- 17
- 18
- 19
- 20
- 21
- 22
- 23
- 24
- 25
- 26
- 27
- 28
- 29
- 30
- 31

LOGIC Hidden unless: #128 Question "**What day of the month were you vaccinated?**" is one of the following answers ("1","2","3","4","5","6","7","8","9","10","11","12","13","14","15","16","17","18","19","20","21","22","23","24","25","26","27","28","29","30","31")

129. **How certain are you of your vaccination date?** *

- Very certain
- Certain +/- 3 days
- Not certain

LOGIC Show/hide trigger exists. Hidden unless: #118 Question "

Have you been vaccinated against COVID-19?

" is one of the following answers ("Yes, two shots","Yes, three shots")

130. **What was your overall experience with your second vaccine dose?**

- No side-effects
- I had some mild symptoms but they didn’t interfere with my activities
- I had symptoms that interfered with my activities
- I had symptoms that prevented me from my activities
- I had to get emergency care or go to hospital for my symptoms

LOGIC Show/hide trigger exists. Hidden unless: #130 Question "**What was your overall experience with your second vaccine dose?** " is one of the following answers ("I had some mild symptoms but they didn’t interfere with my activities","I had symptoms that interfered with my activities","I had symptoms that prevented me from my activities","I had to get emergency care or go to hospital for my symptoms")

131. **Which symptoms did you experience from your second vaccine dose?** (check all that apply)

- ☐ Changes in periods / cycles
- ☐ Pain at the injection site
- ☐ Fever
- ☐ Dry cough
- ☐ Tiredness
- ☐ Aches and pains
- ☐ Sore throat
- ☐ Diarrhea
- ☐ Conjunctivitis
- ☐ Headache
- ☐ Loss of taste or smell
- ☐ A rash on skin, or discolouration of fingers or toes
- ☐ Difficulty breathing or shortness of breath
- ☐ Chest pain or pressure
- ☐ Loss of speech or movement
- ☐ Other (please specify)

LOGIC Hidden unless: #131 Question "**Which symptoms did you experience from your second vaccine dose?** (check all that apply)" is one of the following answers ("Changes in periods / cycles")

132. **Which of the following menstrual changes did you experience?** (check all that apply)

Bleeding

- [ ] Heavier
- [ ] Lighter
- [ ] Spotting

Cycles (length of time between periods)

- [ ] Shorter
- [ ] Longer
- [ ] Become irregular

Period (bleeding days)

- [ ] Longer
- [ ] Shorter
- [ ] Skip / Stopped

Other

- [ ] Other (please specify)

LOGIC Hidden unless: #118 Question "

Have you been vaccinated against COVID-19?

" is one of the following answers ("Yes, three shots")

133. **Which type of vaccine did you have for third vaccine dose?**

- ○ Pfizer-BioNTech
- ○ Johnson & Johnson
- ○ Moderna
- ○ Oxford-AstraZeneca
- ○ Sinopharm
- ○ Other (please specify)
- ○ I am not sure

LOGIC Show/hide trigger exists. Hidden unless: #118 Question "
Have you been vaccinated against COVID-19?

" is one of the following answers ("Yes, three shots")

134. **When did you get your third dose?**

- Before 2021
- January 2021
- February 2021
- March 2021
- April 2021
- May 2021
- June 2021
- July 2021
- August 2021
- September 2021
- October 2021
- November 2021
- December 2021
- I don't know

LOGIC Show/hide trigger exists. Hidden unless: #134 Question "**When did you get your third dose?**" is one of the following answers ("January 2021","February 2021","March 2021","April 2021","May 2021","June 2021","July 2021","August 2021","September 2021")

135. **What day of the month were you vaccinated?**

- I don't know
- 1
- 2
- 3
- 4
- 5
- 6
- 7
- 8
- 9
- 10
- 11
- 12
- 13
- 14
- 15
- 16
- 17
- 18
- 19
- 20
- 21
- 22
- 23
- 24
- 25
- 26
- 27
- 28
- 29
- 30
- 31

Hidden unless: #135 Question "**What day of the month were you vaccinated?**" is one of the following answers ("1","2","3","4","5","6","7","8","9","10","11","12","13","14","15","16","17","18","19","20","21","22","23","24","25","26","27","28","29","30","31")

136. **How certain are you of your vaccination date?** *

- Very certain
- Certain +/- 3 days
- Not certain

Show/hide trigger exists. Hidden unless: #118 Question "

Have you been vaccinated against COVID-19?

" is one of the following answers ("Yes, three shots")

137. **What was your overall experience with your third vaccine dose?**

- No side-effects
- I had some mild symptoms but they didn't interfere with my activities
- I had symptoms that interfered with my activities
- I had symptoms that prevented me from my activities
- I had to get emergency care or go to hospital for my symptoms

Show/hide trigger exists. Hidden unless: #137 Question "**What was your overall experience with your third vaccine dose?** " is one of the following answers ("I had some mild symptoms but they didn’t interfere with my activities","I had symptoms that interfered with my activities","I had symptoms that prevented me from my activities","I had to get emergency care or go to hospital for my symptoms")

138. **Which symptoms did you experience from your third vaccine dose?** (check all that apply)

- ☐ Changes in periods / cycles
- ☐ Pain at the injection site
- ☐ Fever
- ☐ Dry cough
- ☐ Tiredness
- ☐ Aches and pains
- ☐ Sore throat
- ☐ Diarrhea
- ☐ Conjunctivitis
- ☐ Headache
- ☐ Loss of taste or smell
- ☐ A rash on skin, or discolouration of fingers or toes
- ☐ Difficulty breathing or shortness of breath
- ☐ Chest pain or pressure
- ☐ Loss of speech or movement
- ☐ Other (please specify)

LOGIC Hidden unless: #138 Question "**Which symptoms did you experience from your third vaccine dose?** (check all that apply)" is one of the following answers ("Changes in periods / cycles")

139. **Which of the following menstrual changes did you experience?** (check all that apply)

Bleeding

- [ ] Heavier
- [ ] Lighter
- [ ] Spotting

Cycles (length of time between periods)

- [ ] Shorter
- [ ] Longer
- [ ] Become irregular

Period (bleeding days)

- [ ] Longer
- [ ] Shorter
- [ ] Skip / Stopped

Other

- [ ] Other (please specify)

140. **That's it! Do you have any feedback for us?**

New Hidden Value **Action: Hidden Value**
Value: Populates with the **length of time** since the survey taker started the survey

Supplementary Table 12: Participant characteristics by menstrual cycle phase at first COVID-19 vaccination: un-collapsed variables

Characteristic	N	**Overall** N = 1,474[1]	**Follicular** N = 760[1]	**Luteal** N = 714[1]	p-value[2]
**Age (years)**	1,474				0.660
18-24		344 (23%)	175 (23%)	169 (24%)	
25-34		639 (43%)	338 (44%)	301 (42%)	
35-44		491 (33%)	247 (33%)	244 (34%)	
**BMI category**	1,474				0.237
Healthy weight		706 (48%)	365 (48%)	341 (48%)	
Overweight		339 (23%)	186 (24%)	153 (21%)	
Obese		429 (29%)	209 (28%)	220 (31%)	
**Residence**	1,474				0.224
Australia		88 (6.0%)	39 (5.1%)	49 (6.9%)	
Canada		8 (0.5%)	2 (0.3%)	6 (0.8%)	
United Kingdom		332 (23%)	175 (23%)	157 (22%)	
United States of America		1,046 (71%)	544 (72%)	502 (70%)	
**Ethnicity**	1,474				**0.010**
Asian		91 (6.2%)	51 (6.7%)	40 (5.6%)	
Black		62 (4.2%)	24 (3.2%)	38 (5.3%)	
Hispanic		89 (6.0%)	42 (5.5%)	47 (6.6%)	
Middle Eastern		6 (0.4%)	5 (0.7%)	1 (0.1%)	
Mixed		113 (7.7%)	55 (7.2%)	58 (8.1%)	
Other		18 (1.2%)	6 (0.8%)	12 (1.7%)	
Prefer not to say		8 (0.5%)	8 (1.1%)	0 (0%)	
White		1,087 (74%)	569 (75%)	518 (73%)	
**Education**	1,474				0.253
High school or less		105 (7.1%)	48 (6.3%)	57 (8.0%)	
More than high school		1,369 (93%)	712 (94%)	657 (92%)	
**Income**	1,474				0.881
Lower income		341 (23%)	180 (24%)	161 (23%)	
Middle income		573 (39%)	290 (38%)	283 (40%)	
Higher income		465 (32%)	243 (32%)	222 (31%)	
Don't know		95 (6.4%)	47 (6.2%)	48 (6.7%)	
**Stress**	1,474				0.643
Low		157 (11%)	78 (10%)	79 (11%)	
Average		1,072 (73%)	547 (72%)	525 (74%)	
High		185 (13%)	103 (14%)	82 (11%)	
No Answer		60 (4.1%)	32 (4.2%)	28 (3.9%)	
**Medical conditions**	1,474				0.813
Abnormal Pap Smear		112 (7.6%)	61 (8.0%)	51 (7.1%)	
Diabetes		10 (0.7%)	4 (0.5%)	6 (0.8%)	
Eating Disorder		49 (3.3%)	22 (2.9%)	27 (3.8%)	
Endometriosis		19 (1.3%)	10 (1.3%)	9 (1.3%)	
Gynaecological Cancers		1 (<0.1%)	0 (0%)	1 (0.1%)	
Immunosuppressive Therapy Condition		7 (0.5%)	3 (0.4%)	4 (0.6%)	
Interstitial Cystitis		8 (0.5%)	5 (0.7%)	3 (0.4%)	
Multiple		92 (6.2%)	50 (6.6%)	42 (5.9%)	
None		1,050 (71%)	541 (71%)	509 (71%)	
Other Cancers		8 (0.5%)	3 (0.4%)	5 (0.7%)	
PCOS		40 (2.7%)	25 (3.3%)	15 (2.1%)	
Pelvic Inflammatory Disease		1 (<0.1%)	1 (0.1%)	0 (0%)	
Thyroid		56 (3.8%)	26 (3.4%)	30 (4.2%)	
Uterine Polyps/Fibroids		21 (1.4%)	9 (1.2%)	12 (1.7%)	
**Copper IUD**	1,474				0.821
Copper IUD fitted		142 (9.6%)	75 (9.9%)	67 (9.4%)	
Not fitted		1,332 (90%)	685 (90%)	647 (91%)	
**Smoking status**	1,474				0.160
Never		1,034 (70%)	520 (68%)	514 (72%)	
Rarely		273 (19%)	155 (20%)	118 (17%)	
Often		167 (11%)	85 (11%)	82 (11%)	
**Vaccine**	1,474				0.235

**Characteristic**	**N**	**Overall** N = 1,474[1]	**Follicular** N = 760[1]	**Luteal** N = 714[1]	**p-value**[2]
Johnson & Johnson		80 (5.4%)	37 (4.9%)	43 (6.0%)	
Moderna		427 (29%)	219 (29%)	208 (29%)	
Other		6 (0.4%)	3 (0.4%)	3 (0.4%)	
Oxford-AstraZeneca		105 (7.1%)	65 (8.6%)	40 (5.6%)	
Pfizer-BioNTech		856 (58%)	436 (57%)	420 (59%)	

[1]n (%); Median (Q1, Q3) for continuous variables, [2]Wilcoxon rank-sum test for continuous variables; Chi-square test for categorical variables

Supplementary Table 13: Cox model stepwise selection summary

Model	Variables Added	Events/Param	AIC	C-Index	Inf. Obs.	Max Change %	Stability
Minimal	Age	27.3	1,126.3	0.615	41	100.0	Unstable
Plus vaccine type	Second vaccine date	20.5	1,119.8	0.641	47	340.4	Unstable
**Plus medical condition(s)**	**Medical condition(s)**	**16.4**	**1,121.5**	**0.641**	**59**	**192.1**	**Unstable**
Plus BMI	BMI	11.7	1,124.9	0.646	63	403.3	Unstable

Supplementary Table 14: Interaction tests: Association of cycle-phase at vaccination and any self-reported side effects.

Interaction variable	P-value (raw)	P-value (adj)	Significant?
Age group (per 10 years)	***0.043***	*0.300*	*Nominal (†)*
Residence	0.120	0.839	No
Vax1 type	0.148	1.000	No
Smoking status	0.246	1.000	No
BMI category	0.362	1.000	No
Income category	0.429	1.000	No
Stress level (IQR)	0.623	1.000	No

Detailed results for nominally significant interactions:

Interaction variable	Term	OR (98.3% CI)	Int. P (raw)	Int. P (adj)	Term P (raw)	Term P (adj)
Age group (per 10 years)	Cycle phase: follicular	1.13 (0.73-1.74)	0.043	0.300	0.515	1.000
	Cycle phase: follicular × Age: 18-24 years	*1.97 (0.97-4.01)*	*0.043*	*0.300*	*0.022*	*0.156 †*
	Cycle phase: follicular × Age: 35-44 years	1.00 (0.51-1.95)	0.043	0.300	0.992	1.000

Supplementary Table 15: Interaction tests: Association of cycle-phase at vaccination and severity of self-reported side effects.

Interaction variable	P-value (raw)	P-value (adj)	Significant?
Age group (per 10 years)	0.481	1.000	No
BMI category	0.695	1.000	No
Income category	0.854	1.000	No
Residence	0.900	1.000	No
Smoking status	0.959	1.000	No
Stress level	0.976	1.000	No
Vax1 type	0.984	1.000	No

Supplementary Table 16: Interaction tests: Association of cycle-phase at vaccination and number of self-reported side effects.

Interaction variable	P-value (raw)	P-value (adj)	Significant?
Age group (per 10 years)	0.055	0.388	No
Residence	0.176	1.000	No
BMI category	0.305	1.000	No
Smoking status	0.364	1.000	No
Vax1 type	0.398	1.000	No
Income category	0.661	1.000	No
Stress level	0.800	1.000	No
